# Catalytically Active Snake Venom PLA_2_ Enzymes: An Overview of Its Elusive Mechanisms of Reaction: Miniperspective

**DOI:** 10.1021/acs.jmedchem.3c00097

**Published:** 2023-04-05

**Authors:** Juliana Castro-Amorim, Ana Novo de Oliveira, Saulo Luís Da Silva, Andreimar M. Soares, Ashis K. Mukherjee, Maria João Ramos, Pedro A. Fernandes

**Affiliations:** † LAQV, REQUIMTE, Departamento de Química e Bioquímica, Faculdade de Ciências, Universidade do Porto, Rua do Campo Alegre, s/n, 4169-007 Porto, Portugal; ‡ Laboratory of Biotechnology of Proteins and Bioactive Compounds (LABIOPROT), Oswaldo Cruz Foundation, National Institute of Epidemiology in the Western Amazon (INCT-EpiAmO), Porto Velho, Rondônia 76812-245, Brazil; § Sao Lucas Universitary Center (UniSL), Porto Velho, Rondônia 76805-846, Brazil; ∥ Microbial Biotechnology and Protein Research Laboratory, Department of Molecular Biology and Biotechnology, Tezpur University, Tezpur 784028, Assam, India; ⊥ Division of Life Sciences, Institute of Advanced Studies in Science and Technology, Vigyan Path, Garchuk, Paschim Boragaon, Guwahati 781035, Assam, India

## Abstract

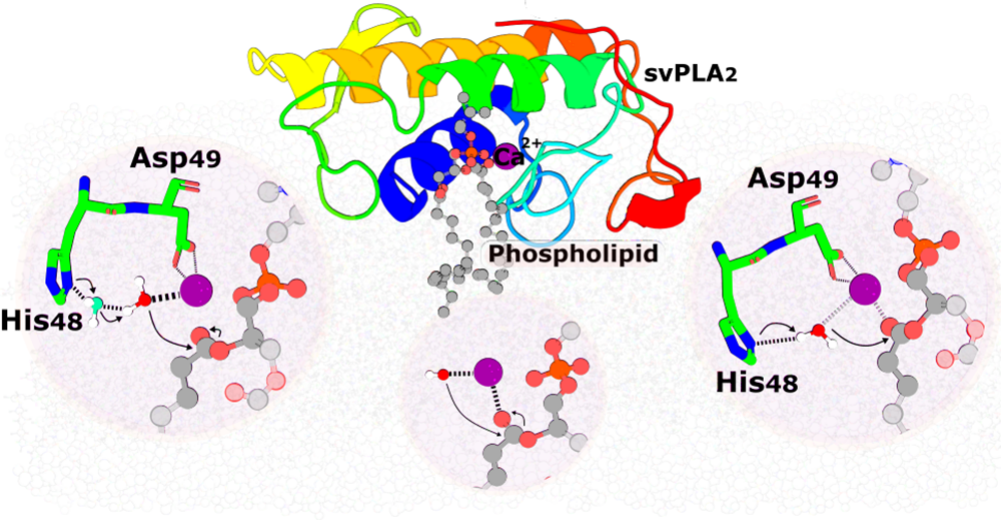

Snake venom-secreted phospholipase A_2_ (svPLA_2_) enzymes, both catalytically active and inactive, are a central component in envenoming. These are responsible for disrupting the cell membrane’s integrity, inducing a wide range of pharmacological effects, such as the necrosis of the bitten limb, cardiorespiratory arrest, edema, and anticoagulation. Although extensively characterized, the reaction mechanisms of enzymatic svPLA_2_ are still to be thoroughly understood. This review presents and analyses the most plausible reaction mechanisms for svPLA_2,_ such as the “single-water mechanism” or the “assisted-water mechanism” initially proposed for the homologous human PLA_2_. All of the mechanistic possibilities are characterized by a highly conserved Asp/His/water triad and a Ca^2+^ cofactor. The extraordinary increase in activity induced by binding to a lipid–water interface, known as “interfacial activation,” critical for the PLA_2_s activity, is also discussed. Finally, a potential catalytic mechanism for the postulated noncatalytic PLA_2_-like proteins is anticipated.

## 1 Introduction to Snake Venom

### 1.1 Epidemiology and Snake Venom Composition: A Brief Account

Snakebite envenomation is a significant public health concern worldwide, particularly in resource-poor regions of tropical and subtropical countries. It is associated with a broad spectrum of pathophysiological effects resulting in high morbidity and mortality. Every year 81 000–138 000 people die from snakebites, and over 400 000 suffer permanent sequelae, such as amputations. The WHO thus recognizes snakebite as the deadliest neglected tropical disease.^1−5^ Administration of animal-derived antivenom remains the more viable available therapy for treating snake envenomation,^6,7^ and early intervention after envenoming is crucial for preventing venom-induced toxicity.^6^ It is particularly relevant for avoiding myotoxicity, a severe condition sometimes coupled with neurotoxicity,^8,9^ consequences of envenomation caused by several snakes of the Elapidae, Viperidae, Atractaspididae, and sometimes Colubridae families.^8−10^ Nevertheless, the antivenom treatment has significant drawbacks: it is costly, needs inpatient administration by trained physicians, refrigerated transport and storage, and its efficacy is limited to the species used in the animal immunization and frequently causes anaphylactic reactions.^5^ Alternatives based on small molecule inhibitors of central venom toxins, such as the svPLA_2_ discussed in this Perspective, are being developed to overcome some of these limitations.^1,11^

Snake venoms are cocktails of bioactive molecules. The venom of each species is unique, and intraspecific variations are common.^1,12^ The snake venoms are mixtures of tenths to hundreds of different components, from which proteins and peptides generally constitute more than 90% of the dry weight.^1^ The enzymes secreted phospholipase A_2_ (svPLA_2_s), metalloproteases, and serine proteases are the most abundant and relevant components in viperid snakes, but there are immense variations.^1^ In Elapid venoms, the most abundant and relevant components are three-finger toxins and svPLA_2_, but the list of variants and exceptions is vast.^1,13,14^

Therefore, snake venoms are a rich source of svPLA_2_ enzymes (EC 3.1.1.4).^1,10^ This enzyme family of enzymes is expressed in almost all venomous snakes,^8^ with Elapidae and Viperidae families exhibiting the highest concentrations and Colubridae (usually nonvenomous) displaying the lowest.^15^

### 1.2 PLA_2_ Enzymes in Snake Venom

**Figure 1:**
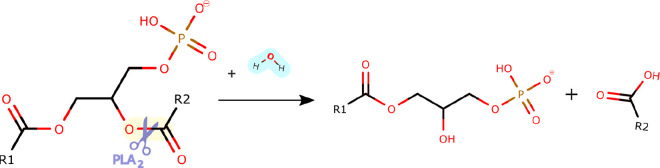
The chemical reaction catalyzed by PLA_2_s. These enzymes act at the *sn*-2 position of glycerophospholipids and hydrolyze the ester bond releasing a lysophospholipid and a free fatty acid. R1 and R2 correspond to the fatty acid tails.

The PLA_2_ superfamily is a broad class of esterases defined by their ability to catalyze the hydrolysis of membrane glycerophospholipids at the *sn*-2 position (Figure 1). The principal contribution of the reaction catalyzed by PLA_2_ is the permeabilization and disruption of the cell membrane integrity, leading to an uncontrolled influx of extracellular molecules, such as Ca^2+^ ions, which trigger a set of events that lead to cell death.^1,16^ In addition, the reaction catalyzed by PLA_2_s releases free fatty acids, such as arachidonic acid and lysophospholipids, which are precursors for several signaling molecules involved in biological processes.^10,17,18^ For example, arachidonic acid can be converted into prostaglandins and leukotrienes, contributing to the mediation of inflammatory processes and pain. On the other hand, the lysophospholipids can be acetylated to platelet-activating factors, which have diverse physiologic roles in the immune system.^17,19^

Over the last decades, significant progress has been made in clarifying the role of the broad PLA_2_ superfamily in many biological functions, particularly in mammals, focusing on human enzymes.^18−21^ These include membrane remodeling, signal transduction, inflammation, antimicrobial defense, metabolism, platelet activation, and cell signaling.^17,18,20,22^ The wide range of biological effects has attracted the interest of many scientists, as PLA_2_s are obvious therapeutic targets for developing pharmaceuticals.^1,17^ However, while the mammalian PLA_2_s enzymes are often nontoxic,^21^ the svPLA_2_s, which are the focus of this Perspective, induce a vast array of toxic effects, including cytotoxicity, hemotoxicity, proinflammatory, (anti)coagulant, and hypotensive effects, besides the above-mentioned myotoxic and neurotoxic effects. These toxic effects may result from catalytic and/or noncatalytic activities.^7,21,23−26^ Although many PLA_2_s discovered in humans have undergone several structural and functional studies, the same does not apply to svPLA_2_, for which our knowledge of the reaction mechanisms is still limited. As a result, this work focuses on the svPLA_2_ potential reaction mechanisms, discussing the current proposals for their chemistry and the arguments supporting each, and drawing comparisons with the proposed reaction for the human PLA_2_.

Based on structural and functional features, the PLA_2_ superfamily is divided into four principal categories: the Ca^2+^-dependent secreted, further subdivided into 17 groups,^27^ the Ca^2+^-dependent cytosolic, the Ca^2+^-independent, and the lipoprotein-associated (LpPLA_2_s) phospholipase A_2_ enzymes.^17,28^ All svPLA_2_ are of the secreted category. The details concerning the remaining types and groups fall out of the scope of this Perspective and can be found in several excellent works on this matter.^18,22^

The svPLA_2_ are small extracellular proteins with a molecular weight of 14–18 kDa, 120–135 amino acid residues, and 6–8 disulfide bridges that contribute to their high degree of stability and a pH optimum at 7.^29,30^ In addition, these enzymes are characterized by a His48/Asp99 dyad at the active site with a Ca^2+^-binding loop and the requirement of mM Ca^2+^ for catalytic activity.^22,27,28^ Besides snake venom, secreted PLA_2_ are found in scorpion and bee venom, among other animal venoms, and in several body fluids as nontoxic enzymes, including blood plasma, pancreatic secretions, seminal fluid, or tears.^19,27^ svPLA_2_ from elapid and viperid snakes share six disulfide bonds and an additional one in a different location on each.^29^

A group numbering system was established based on differences in disulfide bonding patterns, amino acid sequences, molecular weight, and loop insertion^27^ (see Dennis et al.^29^ for a thorough overview of sPLA_2_ classification and history). According to it, snake venoms are divided into two groups of svPLA_2_s, group IA (svPLA_2_-IA), found in snakes of the Elapidae family, and IIA (svPLA_2_-IIA), found in snakes of the Viperidae family.^15,18,31^ The third variant of svPLA_2_ has been found in rear-fanged snakes and classified as group IIE.^15^ However, information about this group is still scarce. Thus, this review will not discuss the group IIE in detail. For example, the venom of the Indian spectacled cobra (*Naja naja,* Elapidae family) belongs to group IA, while the Indian Russell’s Viper svPLA_2_ (*Daboia russelii*, Viperidae family) belongs to group IIA.^32^ Traditionally, the svPLA_2_ of the Gaboon viper (*Bitis gabonica*) is placed separately as the only member of the group-IIB svPLA_2_ due to having only six disulfide bonds. However, other vipers, such as the rhinoceros viper (*Bitis nasicornis*) and the Saharan horned viper (*Cerastes cerastes*), share this characteristic and should be placed in the same svPLA_2_-IIB group.^15^ The nonvenomous mammalian secreted sPLA_2_ that are most similar to the svPLA_2_ are the pancreatic sPLA_2_ (group IB), which have a similar disulfide bond pattern to that of the svPLA_2-_IA and the synovial-specific sPLA_2_ isolated from arthritic synovial fluids and platelets, classified as GPLA_2_-IIA, given their disulfide similarity with the svPLA_2_s -GIIA.^15,27,33^

The svPLA_2_-IAs comprise a single polypeptide chain with 115–125 residues and six disulfide bridges, with an additional one between Cys11 and Cys71 (Renetseder et al.^34^ amino acid numbering). This type of enzyme is ubiquitous in the venom of elapid snakes and accommodates the so-called “elapid loop,” an insertion of 2–3 residues that connects the second α-helix and the β-wing.^15,22^ As an example, the mammalian pancreatic PLA_2_ (PLA_2_-IB) also possesses a loop with five additional amino acid residues at positions 62–67, named as the pancreatic loop.^35,36^

The svPLA_2_-IIA group includes both snake venom and mammalian nonpancreatic PLA_2_. These enzymes comprise 120–125 amino acid residues and six disulfide bonds, with an additional one between Cys133–Cys50. Interestingly, svPLA_2_-IIA enzymes lack the elapid loop present in svPLA_2_-IA. They display, however, an extension of 5–7 amino acid residues at the C-terminal region.^35,36^

## 2 The Structure of svPLA_2_s

**Figure 2:**
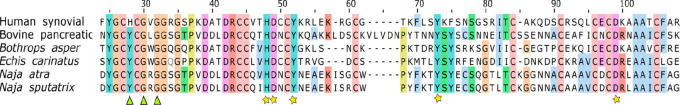
Sequence alignment of six secreted phospholipase A_2_ from different sources: human synovial (UniProtKB AC: P14555), bovine pancreatic (UniProtKB AC: P00593), *B. asper* (UniProtKB AC: P20474), *E. carinatus* (UniProtKB AC: Q7T3S7), *N. atra* (UniProtKB AC: P00598) and *N. sputatrix* (UniProtKB AC: Q92085). Overall, highly conserved residues can be found in the active site, Ca^2+^ binding loop and disulfide bonds. Amino acids residues that have an identity threshold above 40% are colored according to the ClustalX color scheme: hydrophobic (blue), positive charge (red), negative charge (magenta), polar (green), cysteines (pink), glycines (orange), prolines (yellow), aromatic (cyan), and unconserved (white). The numbering shown is that from Renetseder et al.^34^ Green triangles indicate the locations of the residues involved in the calcium-binding, while yellow stars indicate those involved in the active site.

Over 200 nonredundant, complete, and reviewed sequences of svPLA_2_s can be found in the UniProtKB Database (accessed in March).^37^ Their primary structure shows a significant percentage of sequence identity among species, and the X-ray structures deposited in the Protein Data Bank (PDB) (e.g., snake venoms, humans, and bovines) revealed that svPLA_2_ groups have a markedly similar architecture.^27,29,34,38^ A sequence alignment denoting the conservation of the catalytic center of different species of secreted PLA_2_ is shown in Figure 2, and a sequence identity matrix is presented in Supporting Information (SI), Table S1.

**Figure 3:**
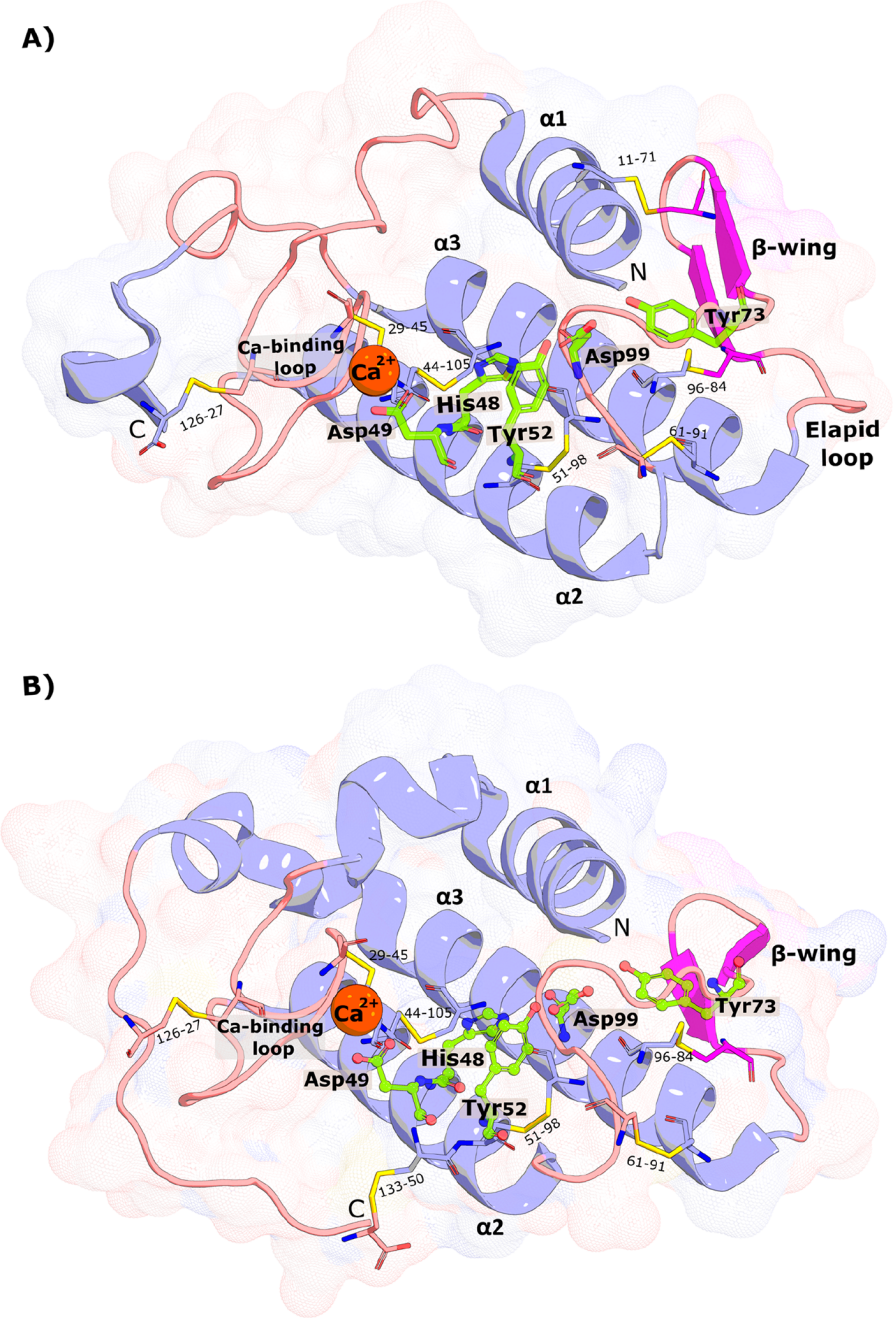
| (A) Structure of the Chinese cobra svPLA_2_-IA (PDB 1POA) and (B) the Indian saw-scaled viper svPLA_2_-IIA (PDB 1OZ6). The active site residues (His48, Asp49, Tyr52, Tyr73, and Asp99) are shown as green sticks, the Ca^2+^ as an orange sphere, and the disulfide bonds as yellow lines. N- and C-terminal regions are also identified. The similarity in the folding is evident. The PyMOL^39^ molecular graphics software package was used to generate the representations.

The svPLA_2_s conserved tertiary structure is mainly characterized by an N-terminal α-helix (α1), two disulfide-connected α-helices (α2 and α3) where the catalytic dyad is placed, a double-stranded antiparallel β-sheet (β-wing), a Ca^2+^-binding loop, and a flexible C-terminal loop (Figure 3). Their folding is also highly similar to the bovine pancreatic and human synovial PLA_2_ (SI, Figure S1).

**Figure 4:**
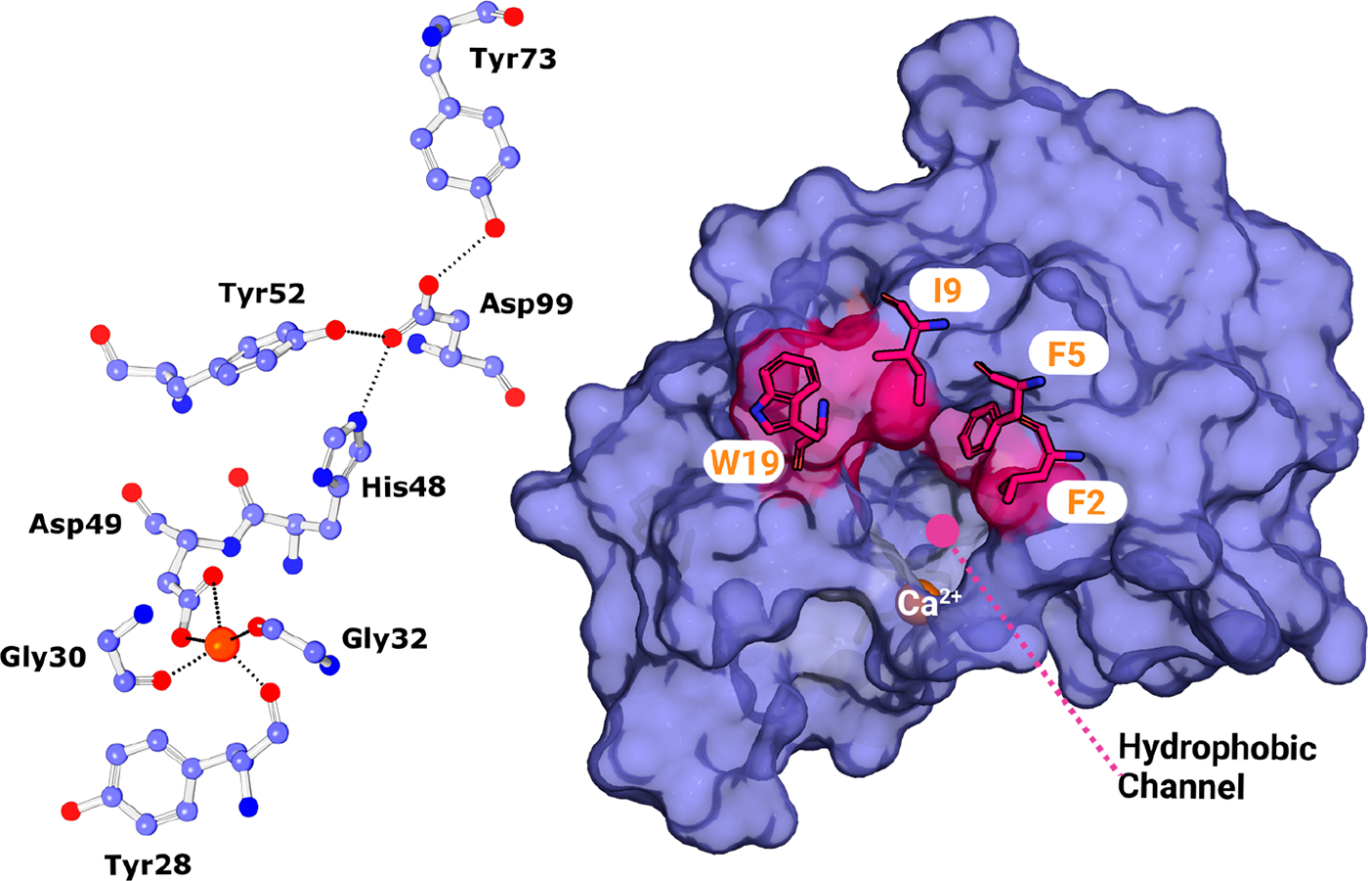
| (left) Ball and stick representation of the residues involved in the catalytic network and respective hydrogen-bonding (dashed lines). (right) Surface representation of the GIA-PLA_2_ isolated from *N. atra* (PDB 1POA) and stick representation of the residues that constitute the hydrophobic channel. The PyMOL molecular graphics software package was used to generate the representations.

In both svPLA_2_ groups, the space between the two antiparallel α-helices is composed of highly hydrophilic solvent-exposed amino acid side chains, and hydrophobic residues are orientated toward the protein’s core. However, the polar residues comprising the catalytic dyad and its hydrogen bond network (His48, Asp49, Tyr52, Tyr73, and Asp99) are buried in the protein core (Figure 4, left).^22,35,40−42^ svPLA_2_s contain an N-terminal α-helix (α1) followed by a short helix. The hydrogen-bonding network formed by these N-terminal α-helices builds a “hydrophobic channel” made by the highly hydrophobic side chains of Leu2, Phe5, Ile9, and Trp19 residues (more common in svPLA_2_-GI) (Figure 4, right) that enhance phospholipid binding while also acting as a shield for His48/Asp99 residues against the surrounding solvent.^31,41,42^

Moreover, it consists of a Ca^2+^-binding loop (Figure 3)^22^ characterized by the carbonyl groups of Tyr28, Gly30, and Gly32, which, together with the β-carboxyl group of Asp49 coordinate the cofactor (Figure 3).^15^ The Tyr28 residue is crucial in binding Ca^2+^ due to the electrostatic interactions between its hydroxyl group oxygen and the Gly35, which result in a greater capacity to bind Ca^2+^ and, thus, a more potent catalytic activity.^22,41^ The distance between the hydroxyl oxygen of Tyr28 and the amino group of Gly35 (around 3.5 Å) is conserved in almost all Asp49 PLA_2_s. This interaction provides the Ca^2+-^binding loop with higher structural stability and a better conformation for metal binding. In agreement, the Asp49Ser svPLA_2_ from *E. carinatus* shows a distorted Ca^2+^-binding loop due to the absence of this interaction.^43^

This loop is followed by the second α-helix (α2), which binds to antiparallel β-sheets cross-linked by disulfide bonds (β-wing region). Following this region is the α-helix 3 (α3), which binds to an exceptionally flexible region, the C-terminal loop, that is believed to be involved in the biological effects of these toxins^22,27^ and allows it to change its conformation and interact with natural lipids.^22,40^ The crystal structures of svPLA_2_-GIA from the Chinese cobra (*Naja atra*) and svPLA_2_-GIIA from the Indian saw-scaled viper (*Echis carinatus*) venom were used to illustrate these structural characteristics (Figure 3). In addition, structures of bovine pancreatic and human synovial PLA_2_ are also shown (SI, Figure S1) to emphasize the above-mentioned similar architecture.

## 3 The Reaction Mechanisms of svPLA_2_

### 3.1 Introduction

Two catalytic mechanisms have been proposed for the family of the secreted PLA_2_s: the “single-water mechanism”^30,44^ and the “assisted-water mechanism”.^45^ The proposals resulted from analyzing X-ray structures of several PLA_2_ from different organisms bound to inhibitors, substrate- and transition-state analogues,^46−50^ results from mutagenesis, and known chemical mechanisms of related enzymes. The crystal structures of about 40 group-I and group-II PLA_2_ from several sources have been determined and deposited in the PDB,^41^ a curated selection of which is given in SI, Table S2. Some have been crystallized with ligands bound in the active site (*holo* form), whereas others were in their *apo* form (SI, Table S2).

The X-ray structures of several svPLA_2_s–inhibitor complexes, three with a transition-state analogue (PDBs 1POB,^48^1POE,^49^ and 1POC(50)) and one with a substrate-derived amide analogue (PDB 5P2P(46)) were fundamental to investigate the mechanisms of reaction.^35^ All of these structures share key properties involved in the catalytic process. According to crystallographic, biochemical, and site-directed mutagenesis studies,^51^ the active site (CCXXH_48_D_49_XC) and the Ca^2+^-binding loop (GCY_28_CG_30_XG_32_GXG) motifs are the most conserved and relevant regions of the PLA_2_ protein.^15^

Mutagenesis also provided fundamental mechanistic information. The most significant is the single-point mutations D49K and D49E, G30S, and H48Q.^52^ Results showed a loss of Ca^2+^ binding and a subsequent loss of enzymatic activity caused by the D49K substitution. Contrarily, the D49E mutant retained the ability to bind Ca^2+^, but this was insufficient for the reaction to occur. This phenomena could be because, with this substitution, the distance between the substrate’s ester bond and the Ca^2+^ ion increases, making it unable to stabilize the tetrahedral intermediate efficiently. These findings unequivocally demonstrate that the side chain carboxylate of Asp49 plays a crucial role in the enzyme’s capacity to bind Ca^2+^.^53,54^ Moreover, the H48Q mutation reduced the enzymatic activity dramatically, and the G30S mutation affected the binding of substrate and Ca^2+^. Thus, the mutagenesis tests pointed out Asp49 and G30 as necessary for Ca^2+^-dependent phospholipid binding and His48 for their hydrolysis, crucial for catalysis.^52−55^

Finally, it was concluded that the conserved His48/Asp99 dyad plus a water molecule constitute a “catalytic triad” similar to the one observed in serine proteases and serine esterases, with the water molecule playing the role of the serine.^56^ In analogy with the serine proteases/esterases, the His48 Nε2 atom appears hydrogen-bonded to the carboxylate Oδ1 atom of Asp99 in X-ray structures.^30,33,41,57^ The hydrogen bond raises the p*K*_a_ of the His48, increasing the basicity of its Nδ1 atom.^33^ Moreover, the p*K*_a_ of the His48 Nδ1 drops from ∼6.5 to 5.5 in the presence of Ca^2+^ ions.^42^

Verheij et al. postulated a catalytic mechanism for the secreted PLA_2_ known as the “single-water mechanism″^44^ based on these findings.^30,42^ Additional validation of the proposed mechanism came later from Scott et al.^30^ based on the structures of secreted PLA2:transition-state analogue complexes.

Later, the study of a bovine pancreatic PLA_2_-GIB cocrystallized with the transition-state analogue 1-hexadecyl-3-(trifluoroethyl)-*sn*-glycero-2-phosphomethanol (MJ33, PDB 1FDK(47)) led to the discovery of a second, Ca^2+^-bound, water molecule, which was proposed to play the role of the nucleophile.^58^ Subsequent studies led Yu et al. to propose an alternative catalytic mechanism, the “assisted-water mechanism”.^45^ In this context, the initial enzyme–substrate complex involves two water molecules at the active site, one that is assisted and Ca^2+^-bound (Ca^2+^-inner coordination sphere). It performs a nucleophilic attack on the substrate and an additional water (Ca^2+^-outer coordination sphere) that acts as a bridge between the attacking water and the His48 base, helping to overcome the considerable distance between the Ca^2+^-bound water proton and the basic His48 Nδ1.

Besides these two mechanisms, it is possible to envisage further alternatives based on the catalytic function of each residue in the mechanisms mentioned above and on known mechanisms from similar enzymes, which involve, for example, a bulk hydroxide ion instead of water as a nucleophile, or the inclusion of additional water in the active center. These mechanisms will also be discussed below.

### 3.2 The Single-Water Mechanism

**Figure 5:**
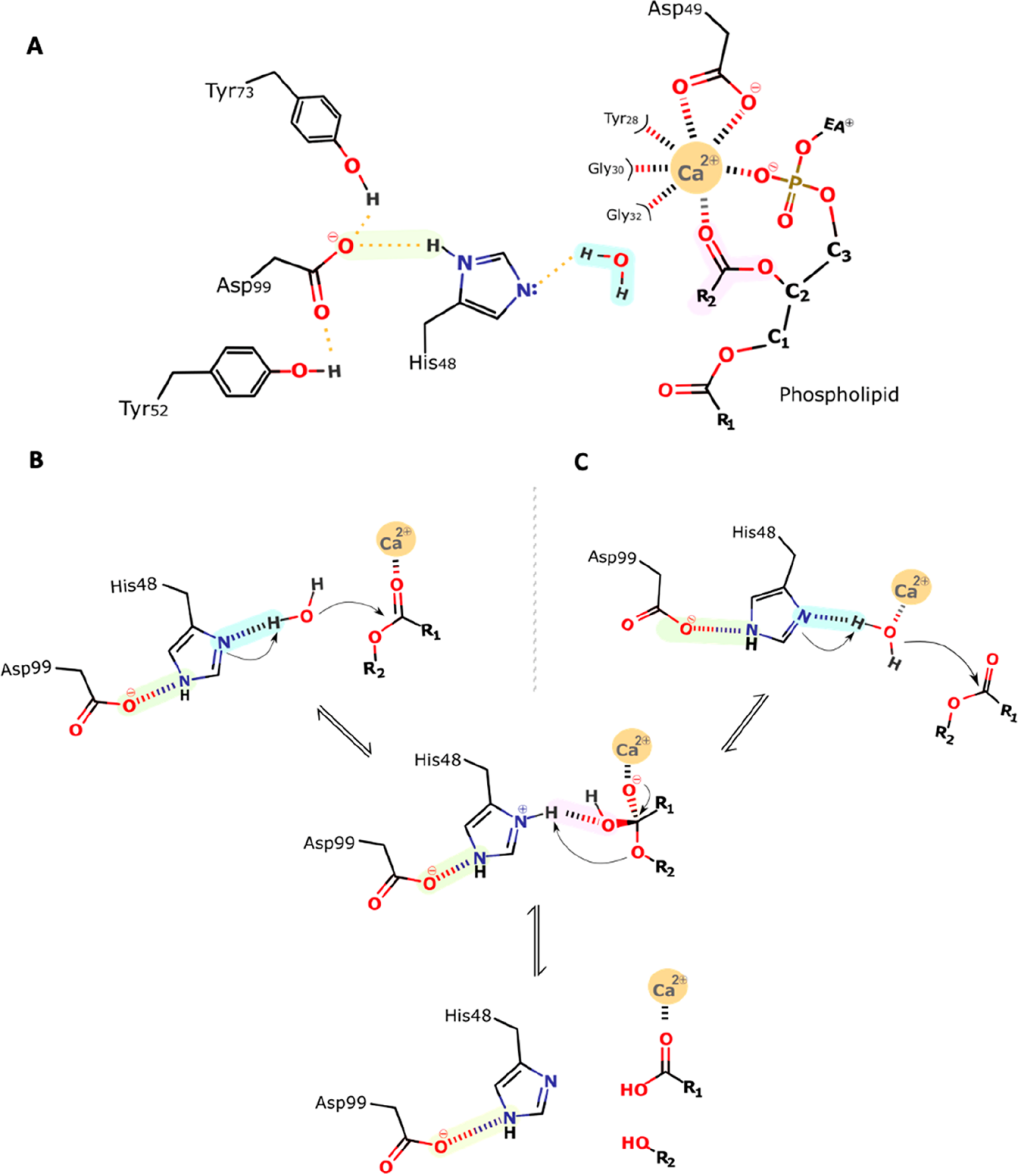
General scheme of the single-water mechanism. (A) The Ca^2+^ coordination shell and the catalytically relevant residues. Their representation will be simplified in the following schemes for simplicity: (B) Verheij proposal,^44^ (C) our proposal, with one calcium-bound water. Step 1: His48 abstracts a proton from the incoming water, which initiates a nucleophilic attack on the *sn*-2 carbonyl carbon of the substrate. Step 2: The produced tetrahedral intermediate oxyanion collapses, eliminating the alkoxyl group, which deprotonates His48. Step 3: Products release; His48 is stabilized by Asp99, which additionally forms hydrogen bonds with Tyr52 and Tyr73. The oxyanion hole that stabilizes the transition state after the nucleophilic attack is formed by the backbone HN group of Gly30 and the Ca^2+^ ion.

According to Verheij (Figure 5), this mechanistic proposal starts with the Ca^2+^ ion hepta-coordinated by a bipyramidal pentagonal cage of oxygen atoms (Figure 5B),^30,35,44^ constituted by the two carboxylate oxygens of the Asp49 side chain, three backbone carbonyl oxygen atoms of the Ca^2+^-binding loop (Tyr28, Gly30, and Gly32),^41,42^ and two water molecules.^35^ The water molecules are displaced upon substrate binding, specifically by the phospholipid phosphate and *sn*-2 carbonyl oxygens.^29^ The former coordination strengthens the substrate’s binding, whereas the latter lowers the reaction activation barrier.^30^

Subsequently, a structurally conserved active-site water molecule is polarized and deprotonated by the His48 Nδ1 atom (Figure 5A), itself polarized by hydrogen bonding of its Nε2 atom to the Asp99 Oδ1 atom. The latter residue stabilizes the cationic form of His48, permitting the deprotonation of water by a formally much less basic species. The formation of a hydroxide ion triggers a nucleophilic attack on the *sn*-2 carbon. This step leads to the formation of a tetrahedral oxyanion intermediate, which is thought to be rate-limiting.^29,30,59^ Moreover, the tetrahedral intermediate’s oxyanion is stabilized by hydrogen bonding to the backbone amine of Gly30 and coordination with the Lewis acid Ca^2+^. The Ca^2+^ cofactor is thought to play a similar role to the Zn^2+^ ion in carboxypeptidases.^42,60^ After the first step, the tetrahedral intermediate collapses by transferring the His48 δNH^+^ proton to the oxyanion of the leaving group.^30,42^ Once the products are released, three water molecules migrate into the active site, from which two coordinate the Ca^2+^ ion and the third replenishes the active cycle for nucleophilic attack of the subsequent turnover.^30,41^

Based on the mechanism of action indicated above, we infer a modified version of the postulated Verheij mechanism (Figure 5B). In our perspective, this new proposal makes more chemical sense and fits the available experimental data as it includes the fundamental catalytic role of the Ca^2+^ cofactor and the residues His48 and Asp99, which are known to be crucial for the mechanism. However, we agree that our modified version of the postulated Verheij mechanism is not directly supported by the X-ray structures available. The issue with the X-ray structures is that few have the Ca^2+^ cofactor cocrystallized and few have a substrate/transition state analogue cocrystallized. Taking the two premises together, around five structures are available (SI, Table S2, *holo* structures). In those structures, the Ca^2+^ is heptacoordinated to residues 28, 30, 32, and 49 (double coordination) and the ligand phosphate (double coordination). As the coordination number of the Ca^2+^ is seven, there is no place for a water molecule. Nevertheless, in bulk solution, the very abundant water molecules can easily replace one of the carbonyl groups of residues 28, 30, or 32, as water is a better ligand for Ca^2+^. Thus, in this alternative, the initial enzyme–substrate complex involves a Ca^2+^-coordinated water molecule instead of a doubly coordinated Asp49. Such a configuration will be more suitable for the reaction to progress as binding to the Ca^2+^ ion lowers the p*K*_a_ of the bound water molecule (in the same way Zn^2+^ does in snake venom metalloproteinases^1,61^ facilitating its deprotonation). Furthermore, the binding of the water molecule with the consequent displacement of a coordination position of Asp49 oxygen can occur through the “carboxylate-shift” mechanism, a well-known and studied phenomenon in Zn^2+^ coordination shells.^62^ A similar mechanism has also been observed for sulfur bound to molybdenum cofactors and denominated as “sulfur shift”.^63^ Alternatively, one of the critical Ca^2+^-binding loop residues may undergo a configurational change, uncoordinating the Ca^2+^ ion. The reaction’s subsequent steps are carried out the same way as in the Verheij proposal.^44^

### 3.3 The Assisted-Water Mechanism

**Figure 6:**
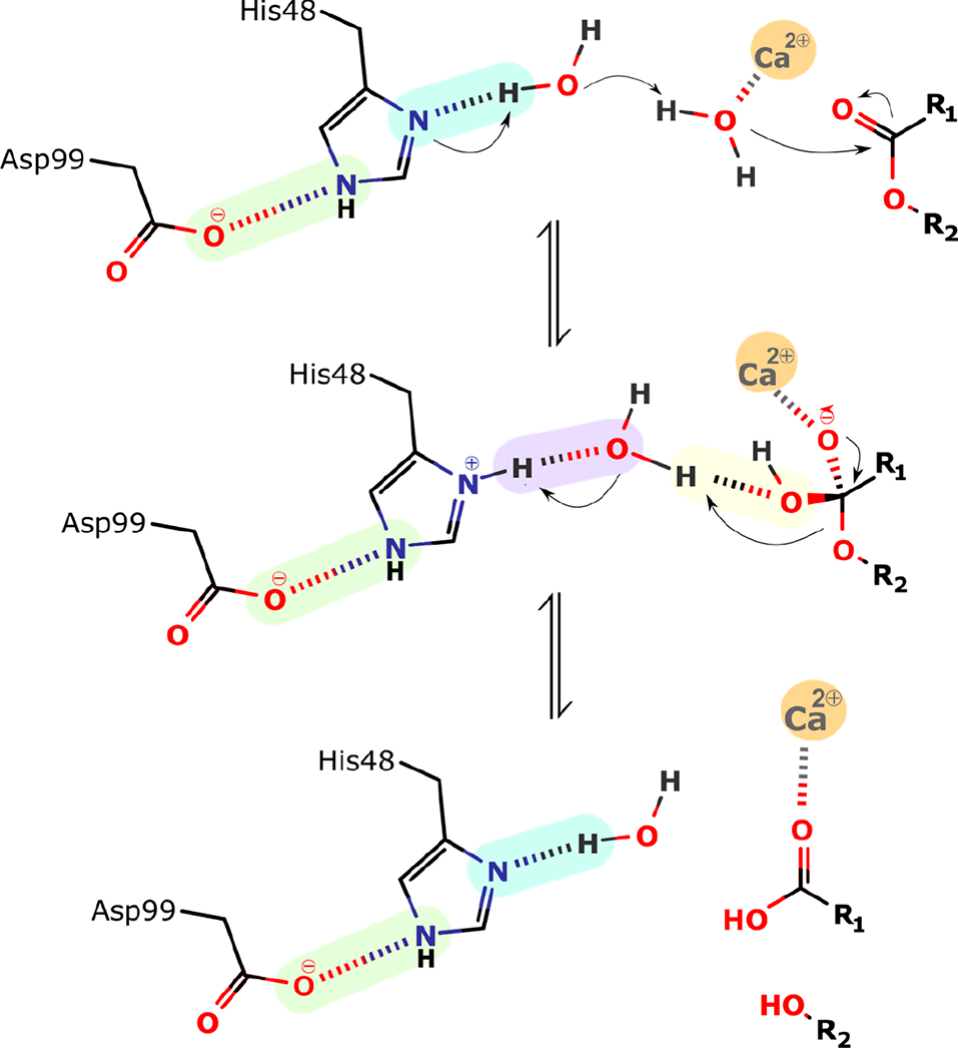
Schematic representation of the assisted-water mechanism proposed by Yu et al.^45^ Step 1: His48, which acts as a general base catalyst, abstracts a proton from the second water, which deprotonates the calcium-bound water molecule. This leads to the nucleophilic attack by the calcium-bound water on the carbonyl carbon of the substrate and the formation of the tetrahedral intermediate. Step 2: The departing alcoholate leaving group is protonated by the second water, which is itself protonated by His 48. Step 3: Collapses and the products are released.

The assisted-water mechanism was proposed by Yu et al.^45^ and is represented in Figure 6. In this proposal, the Ca^2+^ ion coordinates a water molecule and lowers its p*K*_a_. This process is assisted by the hydrogen bond established with the more basic electron pair of Asp49. The nucleophilic Ca^2+^-bound water is hydrogen-bonded to a second water molecule, which, in turn, is hydrogen-bonded to His48. This hydrogen-bonding network overcomes the considerable distance between Ca^2+^ and the His48 Nδ1 atom, corresponding to ca. 6.2 Å.

According to Yu and co-workers, in the first step of the mechanism, the catalytic water molecule deprotonates the second bridging one, itself deprotonated by the Nδ1 atom of the His48, thereby facilitating the reaction (Figure 6). Subsequently, the generated Ca^2+^-bound hydroxide ion water makes a nucleophilic attack on the substrate *sn*-2 carbon atom. This leads to the formation of a tetrahedral intermediate with a Ca^2+^-coordinated oxyanion. During the fallout of the tetrahedral intermediate, His48 protonates the bridging water molecule, which, in turn, protonates the departing alkoxy oxygen.^45^

### 3.4 The Direct Hydroxide Attack Mechanism

Finally, the nucleophilic attack may occur through a hydroxide ion from the bulk water coordinated by the metal cofactor. Divalent metal ions have a high affinity for hydroxide ions. At physiologic pH and temperature (7.4 and 37 °C, respectively), where the hydroxide concentration is ∼56 × 10^7^ lower than water, the entropic cost has been estimated to be around 12.2 kcal·mol^–1^. However, there are known cases where the binding free energy of the hydroxide ion to enzymes divalent cation compensates almost entirely for the high entropic cost of exchanging water by hydroxide into the Ca^2+^ coordination shell, and the lower barrier they provide compensates for the cost of the lower abundance of hydroxide-bound enzymes (2.7 kcal·mol^–1^).^64,65^

**Figure 7:**
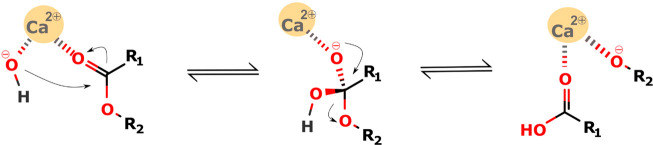
Schematic representation of the hydroxide direct nucleophilic attack mechanism; Step 1: The hydroxide nucleophile attacks at the electrophilic C of the ester C=O leading to the formation of the tetrahedral intermediate. Step 2: The intermediate collapses, reforming the C=O. Step 3: Departure of the alkoxide leaving group, RO^–^.

In this scenario, the Ca^2+^-bound hydroxide attacks the *sn*-2 carbon of the substrate, forming the tetrahedral intermediate (Figure 7). In addition, the Ca^2+^ cofactor stabilizes the C–O^–^ bond by coordinating it. The intermediate then collapses, reconstructing the C=O bonded to the metal ion, resulting in the cleavage of the bond and departure of the alkoxide group (RO^–^). The latter may bind strongly to the Ca^2+^ cofactor, replacing the ionic interaction lost with the hydroxide neutralization. However, the high basicity of the *sn*-2 oxygen makes it a poor leaving group

Nevertheless, the oxyanion can be easily protonated by the much more acidic fatty acid carboxylate, generating two suitable leaving groups. Alternatively, an incoming water molecule can bind the active site and be deprotonated by the *sn*-2 oxygen, easing the elimination of the lysophospholipid and generating the hydroxide ion for the next catalytic cycle. In this variant, a bulk hydroxide ion binding would be needed only for the “initiation” turnover, with the nucleophile for the subsequent turnovers generated by the reaction product.

## 4 The Catalytic Mechanism of the Noncatalytic PLA_2_ Proteins

In addition to the classical sPLA_2_ enzymes, viperid venoms possess several toxins designated as “svPLA_2_-like” proteins or “Lys49 svPLA_2_ homologues” (SI, Figure S2). These proteins share a pervasive sequence identity and folding similarity to the svPLA_2_ enzymes. However, they are essentially catalytically inactive.^66^ The fact that svPLA_2_-like proteins exist exclusively in viperid venoms indicates that they emerged after the Elapidae and Viperidae family divergence.^15^ Intriguingly, while having extremely limited or no enzymatic activity, Lys49-PLA_2_-like enzymes can elicit several pharmacological effects, such as myotoxicity, cytotoxicity, hyperalgesia, and edema-inducing activities, irrespective of their catalytic activity.^67,68^

PLA_2_-like proteins have the Asp49 residue substituted by a Lys or, more rarely, by a Ser, Asn, or Gln residue. Unfortunately, there is not enough data about the Ser/Asn/Gln variants to draw a picture of their catalytic possibilities. It is tempting to speculate that at least the Ser variant might retain some activity, as the Ser-His-Asp constitutes the classical catalytic triad of serine esterases.^56^ However, the correct substrate position and a proper oxyanion hole are mandatory for the reaction, but there is not enough data or model about this system.

As mentioned before, Asp49 is crucial for Ca^2+^ binding and, thus, for the catalytic mechanism (Figures 4 and 5). In addition, the Ca^2+^-binding loop has an open conformation due to the Tyr28Asn mutation, which induces a more extended Asn28–Gly35 interaction.^69^ This loop conformation further impairs the coordination of the Ca^2+^ cofactor.^69^ Thus, the loss of the productive Ca^2+^-binding loop conformation might be a reason why the Lys49 sPLA_2_ cannot bind the Ca^2+^ ion.^69,70^ The lack of Ca^2+^, essential for stabilizing the tetrahedral intermediate, was believed to be why these proteins cannot perform the catalytic cleavage of phospholipids.^69,71^ This theory was backed by structural studies on the svPLA_2_-like enzyme from *A. piscivorus piscivorus,* which revealed that the Lys49 Nε atom was placed in the Ca^2+^ atom position from Asp49-svPLA_2_.^71−73^ However, *in vitro* assays have demonstrated that these variants exhibit catalytic activity, albeit they are minimal.^74,75^ Nevertheless, this observation was contested by others, which attributed the residual catalytic activity of PLA_2_-like proteins to insufficient protein purification and contamination with PLA_2_ enzymes.^53,73,76^

However, experiments by Pedersen et al.^74^ indicated that PLA_2_-like proteins might cleave phospholipids but cannot release the fatty acid product and suggested a covalent binding between the fatty acid and the protein, retaining the fatty acid at the binding site. This hypothesis was later reinforced by the crystallographic structure of a svPLA_2_-like protein denominated PrTX-II, purified from the venom of the viper carpet-jararaca (*Bothrops pirajai)* complexed (albeit noncovalently) with a fatty acid molecule (tridecanoic acid) bound to the active site.^77^

**Figure 8:**
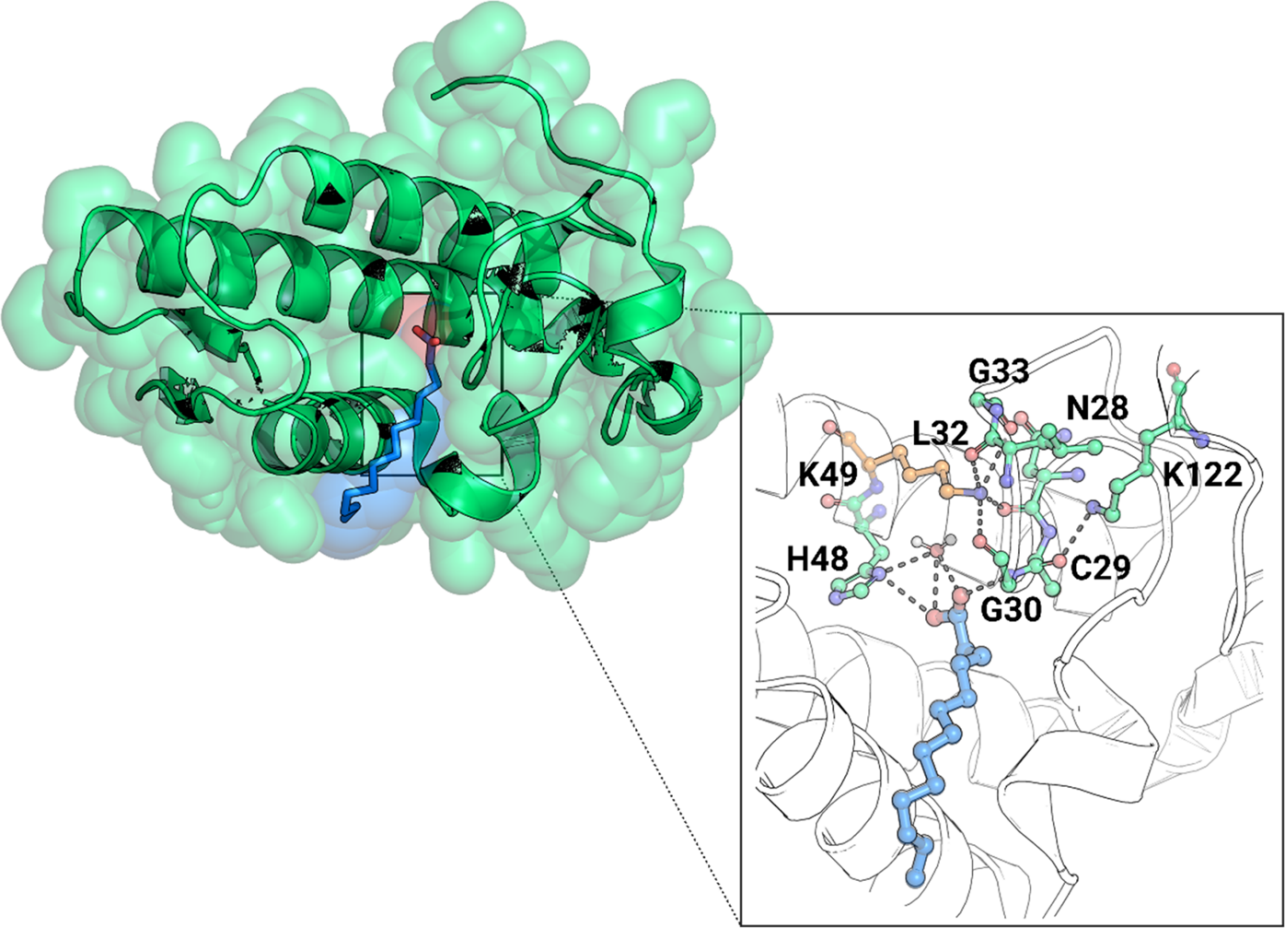
Cartoon representation of the complex between *Bothrops pirajai* PrTX-II and a fatty acid (tridecanoic acid) (PDB 1QLL).^77^ A close-up view of the binding site of PrTX-II, with the fatty acid molecule stabilized by a hydrogen bond with the Cys29–Gly30 peptide bond, which is hyperpolarized by the Lys122, increasing its affinity for the fatty acid. The Ca^2+^ binding loop interactions with the Nε atom of the Lys49 residue are also shown. PyMOL molecular graphics software package was used to generate the representations.

When bound to the Lys49-PLA_2_s active site, the fatty acid molecule is stabilized by a hydrogen bond with the amide group of the Cys29–Gly30 peptide bond (Figure 8).^77^ Furthermore, the interaction between the carbonyl group of Cys29 and the remarkably conserved Lys122 among Lys49-PLA_2_s causes this peptide bond to become hyperpolarized, further strengthening the bond with the fatty acid. In light of these findings, Lee and colleagues hypothesized that the PLA_2_-like limited or absent catalytic activity could be caused by this strong interaction, which increases the affinity for the fatty acid headgroup. Thus, the product’s release from the active site would be blocked at the product release stage of the catalysis cycle, leading to enzyme inhibition.^69,71,72^ In 2005, Ambrosio and colleagues proposed a synergistic activity between these enzymes.^71^ According to them, the PLA_2_s-like proteins bind membrane fatty acids produced by the PLA_2_ enzymes, stabilizing the active conformation of the PLA_2_s-like proteins.^71,78^

Based on the structural and mechanistic evidence described above, we believe that the mechanism by which PLA_2_-like proteins perform phospholipid cleavage is similar to one of the svPLA_2_ enzymes. Thus, an active-site water molecule, whose p*K*_a_ is lowered by hydrogen bonding to Lys49 is deprotonated by His48, which in turn is stabilized by a salt bridge to the Asp99 residue (Renetseder et al.^34^ numbering system) and performs a nucleophilic attack on the phospholipid *sn*-2 bond. The positive charge of the Lys49 side chain amine plays the role of the Ca^2+^ ion by stabilizing the formal negative charge of the transition state oxyanion. Upon collapse from the transition state into the products, the lysophospholipid fragment leaves the active site. Still, the ionic hydrogen bond between the enzyme and the fatty acid carboxylate traps this reaction fragment at the binding site and interrupts the catalytic cycle.^77^

## 5 Interfacial Activation: A Phenomenon That Increases Catalytic Activity

**Figure 9:**
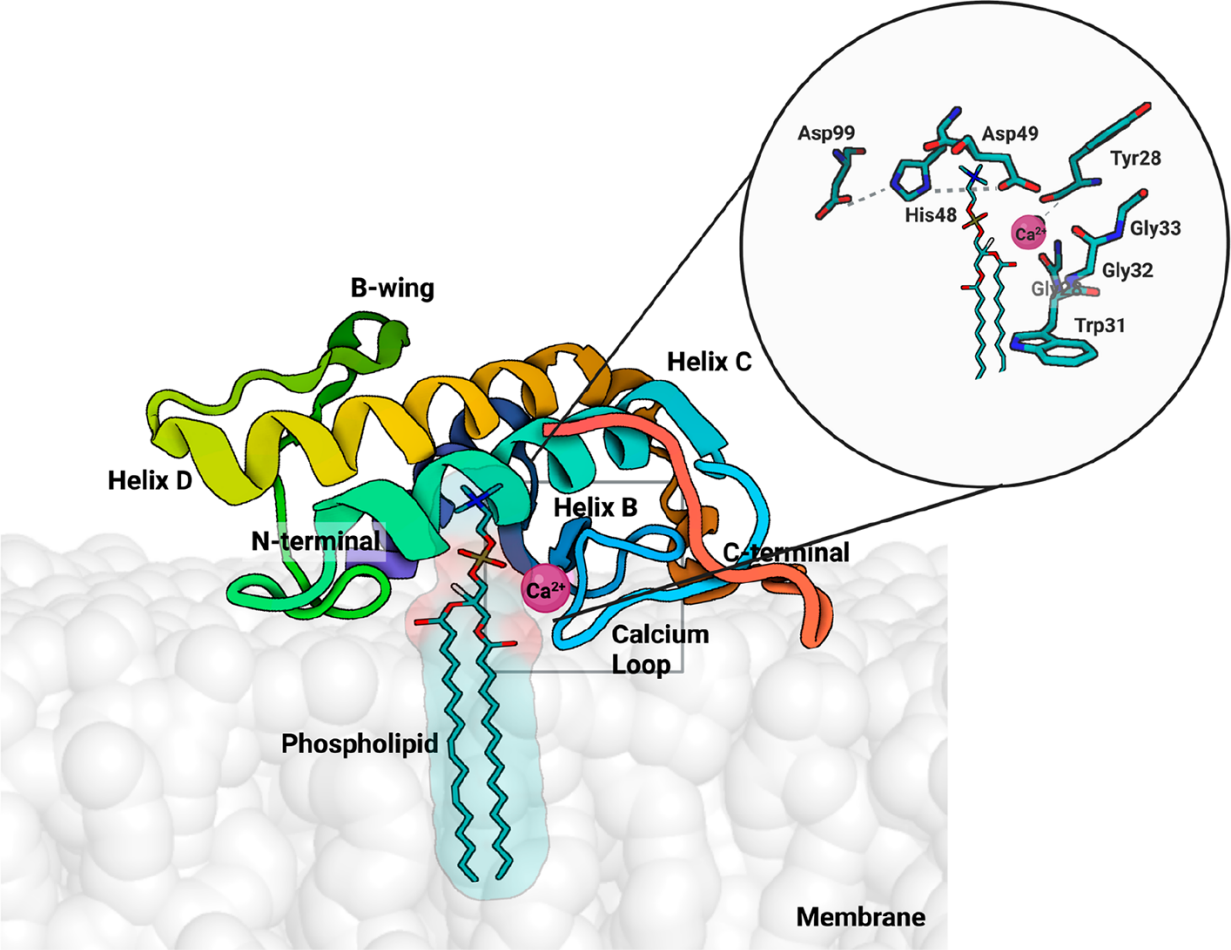
Representation of the svPLA (PDB 5TFV) with the phospholipid bilayer membrane, created using the CHARMM-GUI web interface.^79^ The protein orientation in the membrane was automatically set up with the PPM 2.0 server, and the substrate was manually inserted. In the active center, there is a bound phospholipid substrate in which the color red represents oxygen, phosphorus is brown, nitrogen is blue, and carbon is white; the enzyme is shown in cartoon representation, and the Ca^2+^ ion is shown in dark pink. Binding to the membrane makes the enzyme more active for several orders of magnitude. PyMOL molecular graphics software package was used to generate the representations.

Secreted PLA_2_ enzymes, among which are the svPLA_2_, hydrolyze phospholipids in monomeric, micellar, or lipid bilayer phases.^21^ Except for group III PLA_2_, these enzymes share a phenomenon termed “interfacial activation”,^17,19,21,57^ which has long been captivating the scientific community.^29^ Interfacial activation is the process by which the catalytic activity of secreted PLA_2_ increases dramatically (up to 10 000-fold) when the substrate is shifted from a monomolecular dispersed form to a higher ordered and larger lipid aggregate (Figure 9).^17,19,21,57^ Thus, the protein interaction with large lipid aggregates seems essential for reaching high activity in svPLA_2_.^29,35^ However, the phenomenon’s molecular origin is not understood despite the significant interest and effort.^19,35^

A related phenomenon has been observed in the triglyceride lipase from *Thermomyces lanuginose*.^80^ When the substrate is above its solubility limit, the enzyme is noticeably more active, meaning that its reaction rate is larger toward aggregated than monodispersed substrates. The origin of this effect lies in a lipase conformational rearrangement promoted by contact with lipid aggregates.^51,80^

**Figure 10:**
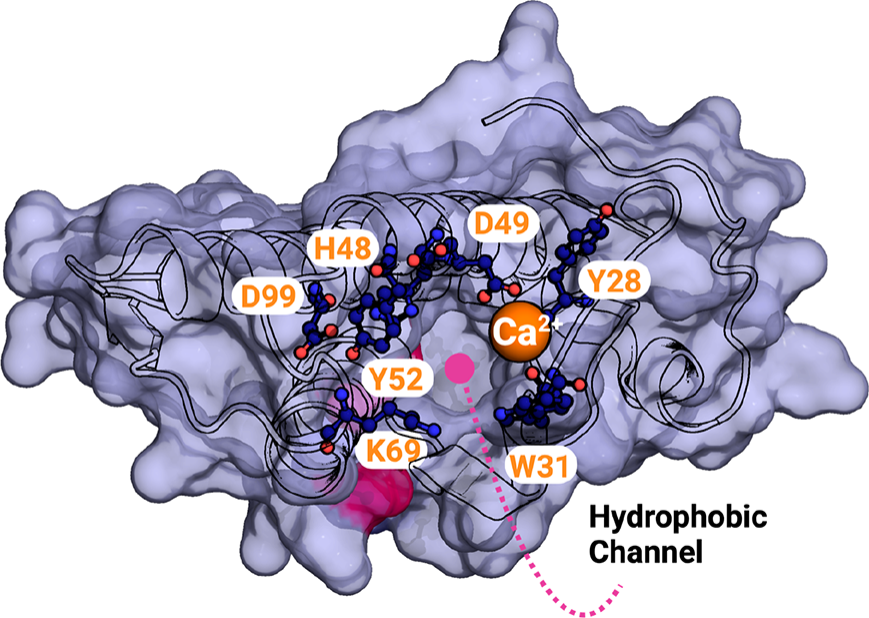
Surface representation of the *E. carinatus* svPLA_2_-IIA (PDB 1OZ6)^81^ hydrophobic channel, with the Trp31 and Lys69 residues in evidence. Catalytic residues are also indicated. The calcium ion is represented as an orange sphere. PyMOL molecular graphics software package was used to generate the representations.

The molecular basis of the interfacial activation of lipases inspired possibilities to explain the same phenomenon in svPLA_2_ enzymes, as binding to the membrane is a fundamental step of the svPLA_2_ reaction cycle. The first step of svPLA_2_ hydrolysis of cell membranes or vesicles is the nonchemical adsorption to a phospholipidic surface. In this process, the enzyme buries ca. 40 phospholipids at the interfacial-adsorption surface (also called the enzyme ″*i-face*″); the adsorption allows substrate binding at the active site to create a Michaelis–Menten complex.^41,52^ This interaction is based on electrostatic and dispersive interactions between basic residues on the PLA_2_*i-face* and anionic phospholipids at the cell membranes or vesicles.^29,35^ The *i-face* includes the region surrounding the hydrophobic channel connected to the active site where highly conserved residues can be found. In svPLA_2_-IIA enzymes, for instance, Trp31 and Lys69 are among these residues, which have been hypothesized to have a significant role in interfacial binding. Trp31 aromatic ring may act as an anchor increasing the enzyme’s affinity for the substrate (Figure 10).^35,81^

Based on this, two models have been proposed: (1) the “substrate model” which assigns the svPLA_2_ interfacial activation to the physical properties of the aggregated substrate that facilitates its diffusion through a hydrophobic channel to the catalytic site,^30^ and (2) the “enzyme model” that suggests an enzymatic conformational change caused by the binding to the membrane interface, with the change being responsible for the enzyme’s interfacial activation.^51^

Concerning the “substrate model”, Scott et al.^30^ proposed that the high lipid ordering may facilitate diffusion to the active site, reducing the conformational entropy cost of phospholipid binding. The phospholipid has many conformations in solution but far less at the active site, implying a high entropic cost for binding. In the membrane, the phospholipid conformational space is much more restricted, reducing the binding entropic penalty. In addition, the conformation of the substrate in the membrane is similar to the one at the svPLA_2_ binding site. Moreover, the tight contact between the enzyme and the phospholipid surface precludes the unfavorable solvation of the tails while migrating from the membrane into the active site. Concerning the “enzyme model” even though the proposal has precedent in the lipase enzyme family, the specific conformational change, leading to a higher catalytic activity is still to be identified. Thus, the proposal lacks supporting evidence at the moment.^51^ An essential aspect of these proposals is that they are not mutually exclusive; both can contribute to the observed increase in reaction rate at aggregated phospholipid surfaces. Therefore, further studies are necessary to fully understand this exciting phenomenon’s molecular nature.

## 6 Conclusions

In this paper, several proposals for the mechanism of action of svPLA_2_ are presented and critically analyzed. In light of the principles of enzyme catalysis drawn from decades of computation in similar enzymes, we propose new mechanistic possibilities that match all experimental data and have precedent in other enzymatic systems. These include a modified version of the Verheij single-water mechanism and a direct hydroxide attack mechanism. The analysis discussed in this review raises the possibility that svPLA_2_s follow a range of different reaction mechanisms, depending on the volatile organization of the solvent around the active site, in particular, because the intrinsic chemistry of the several proposed mechanistic alternatives is similar. For example, the intrinsic difference in the propensity for the “single water” or the “assisted water” mechanism stems from the fine structure of the active site and the distance between the Ca^2+^ ion and His48. Given the well-known plasticity of enzymatic systems, it is expectable that different active site conformations will favor one or the other pathway. Such a scenario has been found before in aspartic proteases.^82^ Furthermore, the activity of the PLA_2_ increases when the substrate is in the form of large aggregates. We review and critically analyze the “substrate model” and “enzyme model” proposals in the literature to explain this phenomenon and rationalize further the potential origin of the interfacial activation.

Over the past decades, significant advances in computational power have allowed the community to get a completer picture of the underlying enzyme catalytic features that are challenging to investigate adequately using experimental methods. Modeling enzyme-catalyzed reaction processes using hybrid quantum mechanics/molecular mechanics (QM/MM) methods has been probably the most promising approach and has been applied to the human PLA_2_s in the 1990s.^83,84^ Future directions for the understanding of the svPLA_2_ catalytic mechanism shall pass through those methodologies, which will be decisive for discovering crucial principles underpinning the PLA_2_s mechanism of action and represent a starting point for effective rational drug design.

## Supporting Information Available

Sequence identity matrix of six different GI/GII sPLA_2_, representation of GI/GII sPLA_2_ tertiary structures, a selection of available GI/GII sPLA_2_ X-ray crystallographic structures in the PDB, and a sequence alignment of four classic and four PLA_2_-like proteins (PDF)

## Abbreviations Used

α: α helix
*i-face*: interfacial-adsorption surface
kDa: kilodaltons
LpPLA_2_s: lipoprotein-associated phospholipase A_2_ enzymes
Lys49-PLA_2_-like: noncatalytic phospholipase A_2_
Prtx-II: piratoxin-II
QM/MM: quantum mechanics/molecular mechanics
svPLA_2_: snake venom secreted phospholipase A_2_
WHO: World Health Organization

## References

[ref1] Oliveira A. L.; Viegas M. F.; da Silva S. L.; Soares A. M.; Ramos M. J.; Fernandes P. A. The chemistry of snake venom and its medicinal potential. Nat. Rev. Chem. 2022, 6, 451 10.1038/s41570-022-00393-7. PMC9185726 35702592

[ref2] Williams D. J.; Faiz M. A.; Abela-Ridder B.; Ainsworth S.; Bulfone T. C.; Nickerson A. D.; Habib A. G.; Junghanss T.; Fan H. W.; Turner M.; Harrison R. A.; Warrell D. A. Strategy for a globally coordinated response to a priority neglected tropical disease: Snakebite envenoming. PLoS Negl. Trop. Dis. 2019, 13, e0007059 10.1371/journal.pntd.0007059. 30789906 PMC6383867

[ref3] Guidelines for the Production, Control and Regulation of Snake Antivenom Immunoglobulins; World Health Organization: Geneva, 2009.

[ref4] Mukherjee A. K.; Mackessy S. P. Prevention and improvement of clinical management of snakebite in Southern Asian countries: a proposed road map. Toxicon 2021, 200, 140–152. 10.1016/j.toxicon.2021.07.008. 34280412

[ref5] Puzari U.; Mukherjee A. K. Recent developments in diagnostic tools and bioanalytical methods for analysis of snake venom: A critical review. Anal. Chim. Acta 2020, 1137, 208–224. 10.1016/j.aca.2020.07.054. 33153604

[ref6] Gutiérrez J. M.; Calvete J. J.; Habib A. G.; Harrison R. A.; Williams D. J.; Warrell D. A. Snakebite envenoming. Nat. Rev. Dis. Primers 2017, 3 (1), 17063 10.1038/nrdp.2017.63. 28905944

[ref7] Kalita B.; Singh S.; Patra A.; Mukherjee A. K. Quantitative proteomic analysis and antivenom study revealing that neurotoxic phospholipase A2 enzymes, the major toxin class of Russell’s viper venom from southern India, shows the least immuno-recognition and neutralization by commercial polyvalent antivenom. Int. J. Biol. Macromol. 2018, 118, 375–385. 10.1016/j.ijbiomac.2018.06.083. 29924981

[ref8] Isbister G. K.; Mirajkar N.; Fakes K.; Brown S. G.; Veerati P. C. Phospholipase A2 (PLA2) as an early indicator of envenomation in Australian elapid snakebites (ASP-27). Biomedicines 2020, 8 (11), 459 10.3390/biomedicines8110459. 33138056 PMC7692658

[ref9] Harris J. B.; Scott-Davey T. Secreted phospholipases A2 of snake venoms: effects on the peripheral neuromuscular system with comments on the role of phospholipases A2 in disorders of the CNS and their uses in industry. Toxins 2013, 5 (12), 2533–2571. 10.3390/toxins5122533. 24351716 PMC3873700

[ref10] Xiao H.; Pan H.; Liao K.; Yang M.; Huang C. Snake Venom PLA(2), a Promising Target for Broad-Spectrum Antivenom Drug Development. Biomed. Res. Int. 2017, 2017, 6592820 10.1155/2017/6592820. 29318152 PMC5727668

[ref11] Puzari U.; Fernandes P. A.; Mukherjee A. K. Advances in the Therapeutic Application of Small-Molecule Inhibitors and Repurposed Drugs against Snakebite. J. Med. Chem. 2021, 64 (19), 13938–13979. 10.1021/acs.jmedchem.1c00266. 34565143

[ref12] Casewell N. R.; Wüster W.; Vonk F. J.; Harrison R. A.; Fry B. G. Complex cocktails: the evolutionary novelty of venoms. Trends Ecol. Evol. 2013, 28 (4), 219–229. 10.1016/j.tree.2012.10.020. 23219381

[ref13] Borges R. J.; Salvador G. H. M.; Campanelli H. B.; Pimenta D. C.; de Oliveira Neto M.; Usón I.; Fontes M. R. M. BthTX-II from Bothrops jararacussu venom has variants with different oligomeric assemblies: An example of snake venom phospholipases A2 versatility. Int. J. Biol. Macromol. 2021, 191, 255–266. 10.1016/j.ijbiomac.2021.09.083. 34547312

[ref14] Ohno M.; Chijiwa T.; Oda-Ueda N.; Ogawa T.; Hattori S. Molecular evolution of myotoxic phospholipases A(2) from snake venom. Toxicon 2003, 42, 841–854. 10.1016/j.toxicon.2003.11.003. 15019486

[ref15] Križaj B. L. a. I. Snake Venom Phospholipase A2 Toxins. In Handbook of Venoms and Toxins of Reptiles, 2nd ed.; Mackessy S. P., Ed.; CRC Press, 2016.

[ref16] Fernández J.; Caccin P.; Koster G.; Lomonte B.; Gutiérrez J. M.; Montecucco C.; Postle A. D. Muscle phospholipid hydrolysis by Bothrops asper Asp49 and Lys49 phospholipase A2 myotoxins – distinct mechanisms of action. FEBS J. 2013, 280 (16), 3878–3886. 10.1111/febs.12386. 23763831

[ref17] Burke J. E.; Dennis E. A. Phospholipase A2 Biochemistry. Cardiovascular Drugs and Therapy 2009, 23 (1), 49–59. 10.1007/s10557-008-6132-9. 18931897 PMC2823292

[ref18] Schaloske R. H.; Dennis E. A. The phospholipase A2 superfamily and its group numbering system. Biochimica et Biophysica Acta (BBA) - Molecular and Cell Biology of Lipids 2006, 1761 (11), 1246–1259. 10.1016/j.bbalip.2006.07.011. 16973413

[ref19] Burke J. E.; Dennis E. A. Phospholipase A2 structure/function, mechanism, and signaling. J. Lipid Res. 2009, 50, S237–S242. 10.1194/jlr.R800033-JLR200. 19011112 PMC2674709

[ref20] Murakami M.; Sato H.; Taketomi Y. Updating Phospholipase A2 Biology. Biomolecules 2020, 10 (10), 1457 10.3390/biom10101457. 33086624 PMC7603386

[ref21] Kini R. M. Excitement ahead: Structure, function and mechanism of snake venom phospholipase A2 enzymes. Toxicon 2003, 42, 827–840. 10.1016/j.toxicon.2003.11.002. 15019485

[ref22] Bitar L.; Jundia D.; Rima M.; Sabatier J.-M.; Fajloun Z. Bee Venom PLA2 Versus Snake Venom PLA2: Evaluation of Structural and Functional Properties. Venoms Toxins 2022, 2, e01012021189841 10.2174/2666121701999210101225032.

[ref23] Kini R. M. Structure–function relationships and mechanism of anticoagulant phospholipase A2 enzymes from snake venoms. Toxicon 2005, 45 (8), 1147–1161. 10.1016/j.toxicon.2005.02.018. 15922780

[ref24] Dutta S.; Sinha A.; Dasgupta S.; Mukherjee A. K. Binding of a Naja naja venom acidic phospholipase A2 cognate complex to membrane-bound vimentin of rat L6 cells: Implications in cobra venom-induced cytotoxicity. Biochim. Biophys. Acta Biomembr. BBA 2019, 1861 (5), 958–977. 10.1016/j.bbamem.2019.02.002. 30776333

[ref25] Mukherjee A. K.; Kalita B.; Thakur R. Two acidic, anticoagulant PLA2 isoenzymes purified from the venom of monocled cobra Naja kaouthia exhibit different potency to inhibit thrombin and factor Xa via phospholipids independent, non-enzymatic mechanism. PLoS One 2014, 9 (8), e101334 10.1371/journal.pone.0101334. 25118676 PMC4131862

[ref26] Doley R.; Mukherjee A. K. Purification and characterization of an anticoagulant phospholipase A2 from Indian monocled cobra (Naja kaouthia) venom. Toxicon 2003, 41 (1), 81–91. 10.1016/S0041-0101(02)00213-1. 12467665

[ref27] Peggion C.; Tonello F. Short Linear Motifs Characterizing Snake Venom and Mammalian Phospholipases A2. Toxins 2021, 13 (4), 290 10.3390/toxins13040290. 33923919 PMC8073766

[ref28] Murakami M. Novel functions of phospholipase A(2)s: Overview. Biochim. Biophys. Acta Mol. Cell Biol. Lipids 2019, 1864 (6), 763–765. 10.1016/j.bbalip.2019.02.005. 30769093

[ref29] Dennis E. A.; Cao J.; Hsu Y.-H.; Magrioti V.; Kokotos G. Phospholipase A2 Enzymes: Physical Structure, Biological Function, Disease Implication, Chemical Inhibition, and Therapeutic Intervention. Chem. Rev. 2011, 111 (10), 6130–6185. 10.1021/cr200085w. 21910409 PMC3196595

[ref30] Scott D. L.; White S. P.; Otwinowski Z.; Yuan W.; Gelb M. H.; Sigler P. B. Interfacial catalysis: the mechanism of phospholipase A2. Science 1990, 250 (4987), 1541–1546. 10.1126/science.2274785. 2274785 PMC3443688

[ref31] Hiu J. J.; Yap M. K. K. Cytotoxicity of snake venom enzymatic toxins: phospholipase A2 and l-amino acid oxidase. Biochem. Soc. Trans. 2020, 48 (2), 719–731. 10.1042/BST20200110. 32267491 PMC7200639

[ref32] Kumar M. S.; Amjesh R A.; Bhaskaran S.; Delphin R D; Nair A. S.; Sudhakaran P R Molecular docking and dynamic studies of crepiside E beta glucopyranoside as an inhibitor of snake venom PLA2. J. Mol. Model. 2019, 25, 88 10.1007/s00894-019-3954-2. 30847632

[ref33] Kim R. R.; Chen Z.; Mann T. J.; Bastard K.; Scott K. F.; Church W. B. Structural and functional aspects of targeting the secreted human group IIA phospholipase A2. Molecules 2020, 25, 4459 10.3390/molecules25194459. 32998383 PMC7583969

[ref34] Renetseder R.; Brunie S.; Dijkstra B. W.; Drenth J.; Sigler P. B. A comparison of the crystal structures of phospholipase A2 from bovine pancreas and Crotalus atrox venom. J. Biol. Chem. 1985, 260 (21), 11627–11634. 10.1016/S0021-9258(17)39077-4. 4044572

[ref35] Betzel C.; Singh T. P.; Georgieva D.; Genov N. Phospholipase A2. In Handbook of Metalloproteins; Wiley, 2004. 10.1002/0470028637.met055

[ref36] Davidson F. F.; Dennis E. A. Evolutionary relationships and implications for the regulation of phospholipase A2 from snake venom to human secreted forms. J. Mol. Evol. 1990, 31 (3), 228–238. 10.1007/BF02109500. 2120459

[ref37] The Universal Protein Resource (UniProt). Nucleic Acids Res. 2007, 36, D190–D195. 10.1093/nar/gkm895. 18045787 PMC2238893

[ref38] Dijkstra B. W.; Kalk K. H.; Hol W. G. J.; Drenth J. Structure of bovine pancreatic phospholipase A2 at 1.7 Å resolution. J. Mol. Biol. 1981, 147 (1), 97–123. 10.1016/0022-2836(81)90081-4. 7265241

[ref39] The PyMOL Molecular Graphics System; DeLano Scientific: San Carlos, CA, 2002; http://www.pymol.org.

[ref40] Corrêa E. A.; Kayano A. M.; Diniz-Sousa R.; Setúbal S. S.; Zanchi F. B.; Zuliani J. P.; Matos N. B.; Almeida J. R.; Resende L. M.; Marangoni S.; et al. Isolation, structural and functional characterization of a new Lys49 phospholipase A2 homologue from Bothrops neuwiedi urutu with bactericidal potential. Toxicon 2016, 115, 13–21. 10.1016/j.toxicon.2016.02.021. 26927324

[ref41] Zambelli V. O.; Picolo G.; Fernandes C. A. H.; Fontes M. R. M.; Cury Y. Secreted Phospholipases A2 from Animal Venoms in Pain and Analgesia. Toxins 2017, 9 (12), 406 10.3390/toxins9120406. 29311537 PMC5744126

[ref42] Verheij H.; Slotboom A.; De Haas G. Structure and function of phospholiphase A2. In Reviews of Physiology, Biochemistry and Pharmacology; Springer: Berlin, Heidelberg, 1981; pp 91–203. 10.1007/3-540-10961-7_3 7031820

[ref43] Zhou X.; Tan T.-C.; Valiyaveettil S.; Go M. L.; Manjunatha Kini R.; Velazquez-Campoy A.; Sivaraman J. Structural Characterization of Myotoxic Ecarpholin S From Echis carinatus Venom. Biophys. J. 2008, 95 (7), 3366–3380. 10.1529/biophysj.107.117747. 18586854 PMC2547436

[ref44] Verheij H. M.; Volwerk J.; Jansen E.; Puyk W.; Dijkstra B.; Drenth J.; De Haas G. Methylation of histidine-48 in pancreatic phospholipase A2. Role of histidine and calcium ion in the catalytic mechanism. Biochemistry 1980, 19 (4), 743–750. 10.1021/bi00545a021. 7356955

[ref45] Yu B.-Z.; Rogers J.; Nicol G. R.; Theopold K. H.; Seshadri K.; Vishweshwara S.; Jain M. K. Catalytic significance of the specificity of divalent cations as KS* and k cat* cofactors for secreted phospholipase A2. Biochemistry 1998, 37 (36), 12576–12587. 10.1021/bi9728607. 9730830

[ref46] Thunnissen M. M. G. M.; Ab E.; Kalk K. H.; Drenth J.; Dijkstra B. W.; Kuipers O. P.; Dijkman R.; de Haas G. H.; Verheij H. M. X-ray structure of phospholipase A2 complexed with a substrate-derived inhibitor. Nature 1990, 347 (6294), 689–691. 10.1038/347689a0. 2215698

[ref47] Sekar K.; Eswaramoorthy S.; Jain M. K.; Sundaralingam M. Crystal Structure of the Complex of Bovine Pancreatic Phospholipase A2 with the Inhibitor 1-Hexadecyl-3-(trifluoroethyl)-sn-glycero-2-phosphomethanol. Biochemistry 1997, 36 (46), 14186–14191. 10.1021/bi971370b. 9369492

[ref48] White S. P.; Scott D. L.; Otwinowski Z.; Gelb M. H.; Sigler P. B. Crystal structure of cobra-venom phospholipase A2 in a complex with a transition-state analogue. Science 1990, 250 (4987), 1560–1563. 10.1126/science.2274787. 2274787

[ref49] Scott D. L.; White S. P.; Browning J. L.; Rosa J. J.; Gelb M. H.; Sigler P. B. Structures of free and inhibited human secretory phospholipase A2 from inflammatory exudate. Science 1991, 254 (5034), 1007–1010. 10.1126/science.1948070. 1948070

[ref50] Scott D. L.; Otwinowski Z.; Gelb M. H.; Sigler P. B. Crystal structure of bee-venom phospholipase A2 in a complex with a transition-state analogue. Science 1990, 250 (4987), 1563–1566. 10.1126/science.2274788. 2274788

[ref51] Berg O. G.; Gelb M. H.; Tsai M.-D.; Jain M. K. Interfacial enzymology: the secreted phospholipase A2-paradigm. Chem. Rev. 2001, 101 (9), 2613–2654. 10.1021/cr990139w. 11749391

[ref52] Rouault M.; Rash L. D.; Escoubas P.; Boilard E.; Bollinger J.; Lomonte B.; Maurin T.; Guillaume C.; Canaan S.; Deregnaucourt C.; et al. Neurotoxicity and other pharmacological activities of the snake venom phospholipase A2 OS2: the N-terminal region is more important than enzymatic activity. Biochemistry 2006, 45 (18), 5800–5816. 10.1021/bi060217r. 16669624 PMC2796912

[ref53] van den Bergh C. J.; Slotboom A. J.; Verheij H. M.; De Haas G. H. The role of aspartic acid-49 in the active site of phospholipase A2: A site-specific mutagenesis study of porcine pancreatic phospholipase A2 and the rationale of the enzymatic activity of [Iysine49] phospholipase A2 from Agkistrodon piscivorus piscivorus venom. Eur. J. Biochem. 1988, 176, 353–357. 10.1111/j.1432-1033.1988.tb14288.x. 3046944

[ref54] Li Y.; Yu B.-Z.; Zhu H.; Jain M. K.; Tsai M.-D. Phospholipase A2 engineering. Structural and functional roles of the highly conserved active site residue aspartate-49. Biochemistry 1994, 33 (49), 14714–14722. 10.1021/bi00253a009. 7993900

[ref55] Sekar K.; Yu B.-Z.; Rogers J.; Lutton J.; Liu X.; Chen X.; Tsai M.-D.; Jain M. K.; Sundaralingam M. Phospholipase A2 Engineering. Structural and Functional Roles of the Highly Conserved Active Site Residue Aspartate-99. Biochemistry 1997, 36 (11), 3104–3114. 10.1021/bi961576x. 9115986

[ref56] Hedstrom L. Serine Protease Mechanism and Specificity. Chem. Rev. 2002, 102 (12), 4501–4524. 10.1021/cr000033x. 12475199

[ref57] Kang T.; Georgieva D.; Genov N.; Murakami M.; Sinha M.; Kumar R.; Kaur P.; Kumar S.; Dey S.; Sharma S.; et al. Enzymatic toxins from snake venom: Structural characterization and mechanism of catalysis. FEBS J. 2011, 278, 4544–4576. 10.1111/j.1742-4658.2011.08115.x. 21470368

[ref58] Petrova S.; Atanasov V.; Balashev K. Vipoxin and Its Components: Structure–Function Relationship. Adv. Protein Chem. Struct. Biol. 2012, 87, 117–153. 10.1016/B978-0-12-398312-1.00005-6. 22607754

[ref59] Peters A. R.; Dekker N.; Van den Berg L.; Boelens R.; Kaptein R.; Slotboom A. J.; De Haas G. H. Conformational changes in phospholipase A2 upon binding to micellar interfaces in the absence and presence of competitive inhibitors. A proton and nitrogen-15 NMR study. Biochemistry 1992, 31 (41), 10024–10030. 10.1021/bi00156a023. 1390760

[ref60] Christianson D. W.; Lipscomb W. N. Carboxypeptidase A. Acc. Chem. Res. 1989, 22, 62–69. 10.1021/ar00158a003.

[ref61] Lingott T.; Schleberger C.; Gutierrez J. M.; Merfort I. High-resolution crystal structure of the snake venom metalloproteinase BaP1 complexed with a peptidomimetic: insight into inhibitor binding. Biochemistry 2009, 48 (26), 6166–6174. 10.1021/bi9002315. 19485419

[ref62] Sousa S. F.; Fernandes P. A.; Ramos M. J. The Carboxylate Shift in Zinc Enzymes: A Computational Study. J. Am. Chem. Soc. 2007, 129 (5), 1378–1385. 10.1021/ja067103n. 17263422

[ref63] Cerqueira N. M. F. S. A.; Fernandes P. A.; Gonzalez P. J.; Moura J. J. G.; Ramos M. J. The Sulfur Shift: An Activation Mechanism for Periplasmic Nitrate Reductase and Formate Dehydrogenase. Inorg. Chem. 2013, 52 (19), 10766–10772. 10.1021/ic3028034. 24066983

[ref64] Ribeiro A. J. M.; Ramos M. J.; Fernandes P. A. The Catalytic Mechanism of HIV-1 Integrase for DNA 3′-End Processing Established by QM/MM Calculations. J. Am. Chem. Soc. 2012, 134 (32), 13436–13447. 10.1021/ja304601k. 22793648

[ref65] Wilson K. A.; Fernandes P. A.; Ramos M. J.; Wetmore S. D. Exploring the Identity of the General Base for a DNA Polymerase Catalyzed Reaction Using QM/MM: The Case Study of Human Translesion Synthesis Polymerase η. ACS Catal. 2019, 9 (3), 2543–2551. 10.1021/acscatal.8b04889.

[ref66] Maraganore J. M.; Merutka G.; Cho W.; Welches W.; Kezdy F.; Heinrikson R. A new class of phospholipases A2 with lysine in place of aspartate 49. Functional consequences for calcium and substrate binding. J. Biol. Chem. 1984, 259 (22), 13839–13843. 10.1016/S0021-9258(18)89822-2. 6438084

[ref67] Francis B.; Gutierrez J. M.; Lomonte B.; Kaiser I. I. Myotoxin II from Bothrops asper (terciopelo) venom is a lysine-49 phospholipase A2. Arch. Biochem. Biophys. 1991, 284 (2), 352–359. 10.1016/0003-9861(91)90307-5. 1899180

[ref68] Chacur M.; Longo I.; Picolo G.; Gutiérrez J. M.; Lomonte B.; Guerra J.; Teixeira C. d. F. P.; Cury Y. Hyperalgesia induced by Asp49 and Lys49 phospholipases A2 from Bothrops asper snake venom: pharmacological mediation and molecular determinants. Toxicon 2003, 41 (6), 667–678. 10.1016/S0041-0101(03)00007-2. 12727271

[ref69] Fernandes C. A. H.; Marchi-Salvador D. P.; Salvador G. M.; Silva M. C. O.; Costa T. R.; Soares A. M.; Fontes M. R. M. Comparison between apo and complexed structures of bothropstoxin-I reveals the role of Lys122 and Ca2+-binding loop region for the catalytically inactive Lys49-PLA2s. J. Struct. Biol. 2010, 171 (1), 31–43. 10.1016/j.jsb.2010.03.019. 20371382

[ref70] Corrêa L. C.; Marchi-Salvador D. P.; Cintra A. C.; Sampaio S. V.; Soares A. M.; Fontes M. R. Crystal structure of a myotoxic Asp49-phospholipase A2 with low catalytic activity: insights into Ca2+-independent catalytic mechanism. Biochim. Biophys. Acta Proteins Proteom. 2008, 1784 (4), 591–599. 10.1016/j.bbapap.2008.01.007. 18261474

[ref71] Ambrosio A. L. B.; Nonato M. C.; de Araújo H. S. S.; Arni R.; Ward R. J.; Ownby C. L.; de Souza D. H. F.; Garratt R. C. A Molecular Mechanism for Lys49-Phospholipase A2 Activity Based on Ligand-induced Conformational Change*. J. Biol. Chem. 2005, 280 (8), 7326–7335. 10.1074/jbc.M410588200. 15596433

[ref72] Watanabe L.; Soares A. M.; Ward R. J.; Fontes M. R. M.; Arni R. K. Structural insights for fatty acid binding in a Lys49-phospholipase A2: crystal structure of myotoxin II from Bothrops moojeni complexed with stearic acid. Biochimie 2005, 87 (2), 161–167. 10.1016/j.biochi.2004.11.005. 15760708

[ref73] Scott D. L.; Achari A.; Vidal J. C.; Sigler P. B. Crystallographic and biochemical studies of the (inactive) Lys-49 phospholipase A2 from the venom of Agkistridon piscivorus piscivorus. J. Biol. Chem. 1992, 267 (31), 22645–22657. 10.1016/S0021-9258(18)41721-8. 1429612

[ref74] Pedersen J.; Lomonte B.; Massoud R.; Gubensek F.; Gutiérrez J. M.; Rufini S. Autocatalytic acylation of phospholipase-like myotoxins. Biochemistry 1995, 34 (14), 4670–4675. 10.1021/bi00014a021. 7718570

[ref75] Shimohigashi Y.; Tani A.; Matsumoto H.; Nakashima K.-i.; Yamuguchi Y.; Oda N.; Takano Y.; Kamiya H.-o; Kishino J.; Arita H.; Ohno M. Lysine-49-phospholipases A2 from Trimeresurus flavoviridis venom are membrane-acting enzymes. J. Biochem. 1995, 118, 1037–1044. 10.1093/jb/118.5.1037. 8749324

[ref76] Fernandes C. A. H.; Borges R. J.; Lomonte B.; Fontes M. R. M. A structure-based proposal for a comprehensive myotoxic mechanism of phospholipase A2-like proteins from viperid snake venoms. Biochim. Biophys. Acta Proteins Proteom. 2014, 1844 (12), 2265–2276. 10.1016/j.bbapap.2014.09.015. 25278377

[ref77] Lee W.-H.; da Silva Giotto M. T.; Marangoni S.; Toyama M. H.; Polikarpov I.; Garratt R. C. Structural Basis for Low Catalytic Activity in Lys49 Phospholipases A2A Hypothesis: The Crystal Structure of Piratoxin II Complexed to Fatty Acid. Biochemistry 2001, 40 (1), 28–36. 10.1021/bi0010470. 11141053

[ref78] dos Santos J. I.; Soares A. M.; Fontes M. R. M. Comparative structural studies on Lys49-phospholipases A2 from Bothrops genus reveal their myotoxic site. J. Struct. Biol. 2009, 167 (2), 106–116. 10.1016/j.jsb.2009.04.003. 19401234

[ref79] Wu E. L.; Cheng X.; Jo S.; Rui H.; Song K. C.; Dávila-Contreras E. M.; Qi Y.; Lee J.; Monje-Galvan V.; Venable R. M.; et al. CHARMM-GUI Membrane Builder toward realistic biological membrane simulations. J. Comput. Chem. 2014, 35 (27), 1997–2004. 10.1002/jcc.23702. 25130509 PMC4165794

[ref80] Berg O. G.; Cajal Y.; Butterfoss G. L.; Grey R. L.; Alsina M. A.; Yu B.-Z.; Jain M. K. Interfacial Activation of Triglyceride Lipase from Thermomyces (Humicola) lanuginosa: Kinetic Parameters and a Basis for Control of the Lid. Biochemistry 1998, 37 (19), 6615–6627. 10.1021/bi972998p. 9578545

[ref81] Wang X.-q.; Yang J.; Gui L.-l.; Lin Z.-j.; Chen Y.-c.; Zhou Y.-c. Crystal Structure of an Acidic Phospholipase A2from the Venom ofAgkistrodon halyspallas at 2.0 Å Resolution. J. Mol. Biol. 1996, 255 (5), 669–676. 10.1006/jmbi.1996.0054. 8636969

[ref82] Calixto A. R.; Ramos M. J.; Fernandes P. A. Conformational diversity induces nanosecond-timescale chemical disorder in the HIV-1 protease reaction pathway. Chem. Sci. 2019, 10 (30), 7212–7221. 10.1039/C9SC01464K. 31588289 PMC6677113

[ref83] Waszkowycz B.; Hillier I. H.; Gensmantel N.; Payling D. W. A theoretical study of hydrolysis by phospholipase A2: the catalytic role of the active site and substrate specificity. J. Chem. Soc., Perkin Transactions 2 1990, 7, 1259–1268. 10.1039/p29900001259.

[ref84] Waszkowycz B.; Hillier I. H.; Gensmantel N.; Payling D. W. A combined quantum mechanical/molecular mechanical model of the potential energy surface of ester hydrolysis by the enzyme phospholipase A2. J. Chem. Soc., Perkin Transactions 2 1991, (2), 225–231. 10.1039/p29910000225.

