# Supplementary material for: Catalytically Active Snake Venom PLA2 Enzymes: An Overview of Its Elusive Mechanisms of Reaction: Miniperspective
Source: J Med Chem. 2023 Apr 5;66(8):5364–76. doi: 10.1021/acs.jmedchem.3c00097 (PMC10150362; doi:10.1021/acs.jmedchem.3c00097)
Supplement: Supplementary file 1 — jm3c00097_si_001.pdf [file jm3c00097_si_001.pdf]

Supporting Information

Catalytically Active Snake Venom PLA_2 enzymes: An Overview of Its Elusive Mechanisms of Reaction

Juliana Castro-Amorim,[1] Ana Novo de Oliveira,[1] Saulo Luís Da Silva,[1] Andreimar M. Soares,[2,3] Ashis K. Mukherjee,[4,5] Maria João Ramos,[1] and Pedro A. Fernandes[1*]

[1] LAQV, REQUIMTE, Departamento de Química e Bioquímica, Faculdade de Ciências, Universidade do Porto, Rua do Campo Alegre, s/n, 4169-007 Porto, Portugal

[2] Laboratory of Biotechnology of Proteins and Bioactive Compounds (LABIOPROT), Oswaldo Cruz Foundation, National Institute of Epidemiology in the Western Amazon (INCT-EpiAmO), Porto Velho, Rondônia 76812-245, Brazil

[3] Sao Lucas Universitary Center (UniSL), Porto Velho, Rondônia 76805-846, Brazil

[4] Microbial Biotechnology and Protein Research Laboratory, Department of Molecular Biology and Biotechnology, Tezpur University, Tezpur 784028, Assam, India

[5] Division of Life Sciences, Institute of Advanced Studies in Science and Technology, Vigyan Path, Garchuk, Paschim Boragaon, Guwahati 781035, Assam, India

Email: pafernan@fc.up.pt

Contents of SI file

Table S1. The sequence identity matrix of six different $sPLA_2$ resulted from the multiple sequence alignment of a human synovial (UniProtKB AC: P14555), bovine pancreatic (UniProtKB AC: P00593), *B. asper* (UniProtKB AC: P20474), *E. carinatus* (UniProtKB AC: Q7T3S7), *N. atra* (UniProtKB AC: P00598) and *N. sputatrix* (UniProtKB AC: Q92085). Percent sequence identity matrix was created by ClustalW. [1] Higher percentages are represented with darker colors.

Synovial (GIIA)	Pancreatic (GIB)	*B. asper* (GIIA)	*E. carinatus* (GIIA)	*N. atra* (GIA)	*N. sputatrix* (GIA)	
**100**	37	45	47	35	33	Synovial
	**100**	36	35	54	52	Pancreatic
		**100**	52	33	32	*B. asper*
			**100**	35	33	*E. carinatus*
				**100**	95	*N. atra*
					**100**	*N. sputatrix*

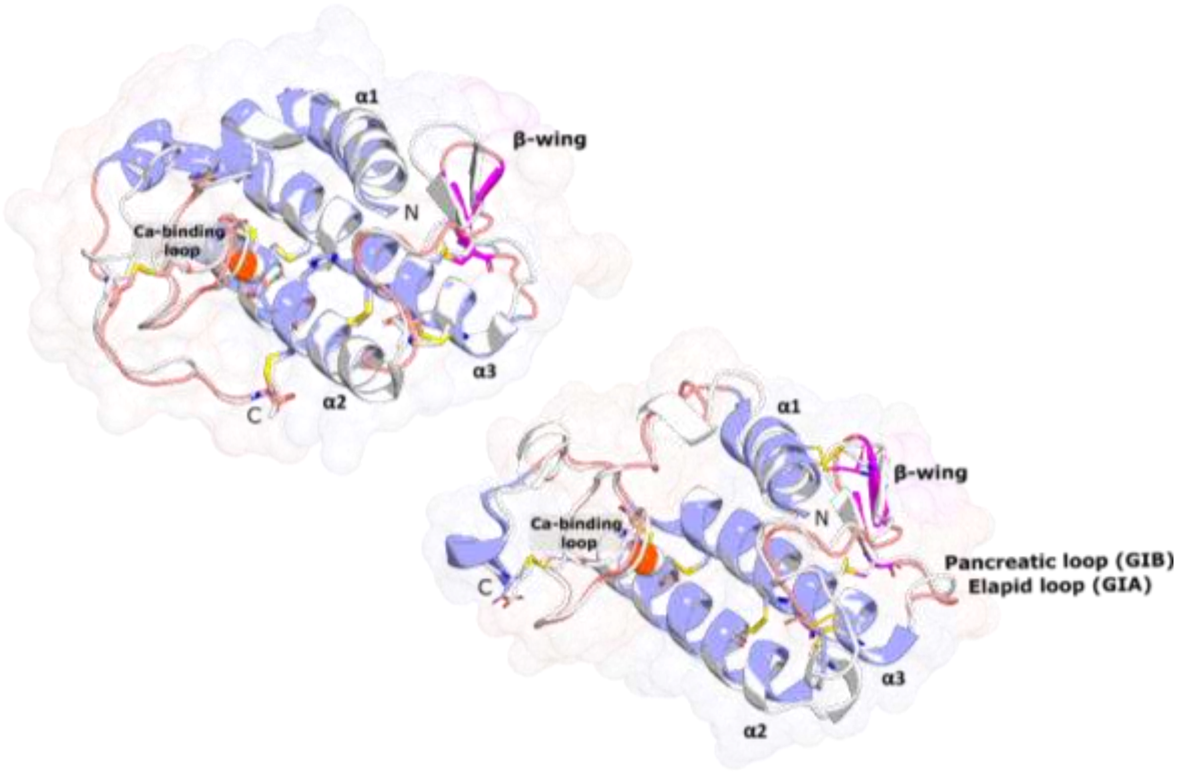

Figure S1. Cartoon representation of PLA_2 tertiary structure. (left) Superimposition of a bovine pancreatic (PDB 1MKV) (white) and snake venom GI-PLA2 from the Chinese cobra (*Naja atra*) (PDB 1POA); (right) Superimposition of a human synovial (white) (PDB 1POE) and snake venom GII-PLA2 from the Indian saw-scaled viper (*Echis carinatus*) (PDB 1OZ6). All PLA2 have highly similar tertiary structures: Helices (lightblue), loops (salmon) and β-sheets (magenta) cartoon. The Ca^{2+} is show as an orange sphere and the disulfide bonds as yellow lines. The Ca^{2+}-binding loop, the N- and C-terminal are also identified. The PyMOL molecular graphics software package was used to generate the representations.

Table S2. A selection of the reported X-ray crystallographic structures of snake venom, human, porcine and bovine GI/GII PLA$_2$.

Group	Source	Species	PDB	Ligands	Resolution	Ref.
**GIA**	Elapid snake venom	*Naja atra*	1POB	holo	2.00 Å	2
**GIA**	Elapid snake venom	*Naja atra*	1POA	apo	1.50 Å	3
**GIB**	Bovine pancreas	*Bos taurus*	1BP2	apo	1.70 Å	4
**GIB**	Bovine pancreas	*Bos taurus*	1MKV	holo	1.89 Å	5
**GIB**	Porcine pancreas	*Sus scrofa*	5P2P	holo	2.40 Å	6
**GIIA**	Human synovial fluid	*Homo sapiens*	1KVO	holo	2.00 Å	7
**GIIA**	Human synovial fluid	*Homo sapiens*	1POE	holo	2.10 Å	8
**GIIA**	Viperid snake venom	*Crotalus atrox*	1PP2	apo	2.50 Å	9
**GIIA**	Viperid snake venom	*Bothrops asper*	5TFV	apo	2.54 Å	10
**GIIA**	Viperid snake venom	*Gloydius halys*	1PSJ	apo	2.00 Å	11
**GIIA**	Viperid snake venom	*Echis carinatus*	1OZ6	apo	2.60 Å	12

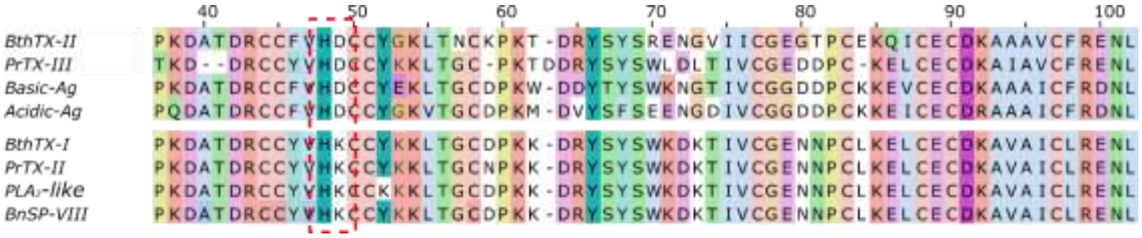

Figure S2. Sequence alignment of four classic PLA$_2$ (BthTX-II, PrTX-III, Basic-PLA$_2$ and Acidic-PLA$_2$ - UniProtKB ACs: P45881, P58464, O42187 and P14418, respectively) and four PLA$_2$-like proteins (BthTX-I, PrTX-II, PLA2-like and BnSP-VIII – UniProtKB AC: Q90249, P82287, I6L8L6 and Q9IAT9, respectively). The replacement of the Asp residue by a Lysine at position 49 is highlighted in a red dashed box. High sequence identity is also evidenced. Amino acids are colored according to the ClustalX color scheme: hydrophobic (blue), positive charge (red), negative charge (magenta), polar (green), cysteines (pink), glycines (orange), prolines (yellow), aromatic (cyan) and unconserved (white). Created with Jalview 2.11 software. [13]

References

(1) Thomopson, J.; Higgins, D. G.; Gibson, T. J. ClustalW. *Nucleic Acids Res* **1994**, *22*, 4673-4680.

(2) White, S. P.; Scott, D. L.; Otwinowski, Z.; Gelb, M. H.; Sigler, P. B. Crystal structure of cobra-venom phospholipase A2 in a complex with a transition-state analogue. *Science* **1990**, *250* (4987), 1560-1563. https://doi.org/10.1126/science.2274787.

(3) Scott, D. L.; White, S. P.; Otwinowski, Z.; Yuan, W.; Gelb, M. H.; Sigler, P. B. Interfacial catalysis: the mechanism of phospholipase A2. *Science* **1990**, *250* (4987), 1541-1546. https://doi.org/10.1126/science.2274785.

(4) Dijkstra, B. W.; Kalk, K. H.; Hol, W. G. J.; Drenth, J. Structure of bovine pancreatic phospholipase A2 at 1.7 Å resolution. *J. Mol. Biol.* **1981**, *147* (1), 97-123. DOI: https://doi.org/10.1016/0022-2836(81)90081-4.

(5) Sekar, K.; Kumar, A.; Liu, X.; Tsai, M. D.; Gelb, M. H.; Sundaralingam, M. Structure of the complex of bovine pancreatic phospholipase A2 with a transition-state analogue. *Acta Crystallogr. D Biol. Crystallogr.* **1998**, *54* (Pt 3), 334-341. https://doi.org/10.1107/s090744499701247x.

(6) Thunnissen, M. M. G. M.; Ab, E.; Kalk, K. H.; Drenth, J.; Dijkstra, B. W.; Kuipers, O. P.; Dijkman, R.; de Haas, G. H.; Verheij, H. M. X-ray structure of phospholipase A2 complexed with a substrate-derived inhibitor. *Nature* **1990**, *347* (6294), 689-691. https://doi.org/10.1038/347689a0.

(7) Cha, S.-S.; Lee, D.; Adams, J.; Kurdyla, J. T.; Jones, C. S.; Marshall, L. A.; Bolognese, B.; Abdel-Meguid, S. S.; Oh, B.-H. High-resolution X-ray crystallography reveals precise binding interactions between human nonpancreatic secreted phospholipase A2 and a highly potent inhibitor (FPL67047XX). *J. Med. Chem.* **1996**, *39* (20), 3878-3881. https://doi.org/10.1021/jm960502g.

(8) Scott, D. L.; White, S. P.; Browning, J. L.; Rosa, J. J.; Gelb, M. H.; Sigler, P. B. Structures of free and inhibited human secretory phospholipase A2 from inflammatory exudate. *Science* **1991**, *254* (5034), 1007-1010. https://doi.org/10.1126/science.1948070.

(9) Brunie, S.; Bolin, J.; Gewirth, D.; Sigler, P. B. The refined crystal structure of dimeric phospholipase A2 at 2.5 A. Access to a shielded catalytic center. *J. Biol. Chem.* **1985**, *260* (17), 9742-9749.

(10) Salvador, G. H. M.; dos Santos, J. I.; Lomonte, B.; Fontes, M. R. M. Crystal structure of a phospholipase A2 from Bothrops asper venom: Insights into a new putative "myotoxic cluster". *Biochimie* **2017**, *133*, 95-102. DOI: https://doi.org/10.1016/j.biochi.2016.12.015.

(11) Wang, X.-q.; Yang, J.; Gui, L.-l.; Lin, Z.-j.; Chen, Y.-c.; Zhou, Y.-c. Crystal Structure of an Acidic Phospholipase A2from the Venom ofAgkistrodon halyspallas at 2.0 Å Resolution. *J. Mol. Biol.* **1996**, *255* (5), 669-676. DOI: https://doi.org/10.1006/jmbi.1996.0054.

(12) Jasti, J.; Paramasivam, M.; Srinivasan, A.; Singh, T. P. Structure of an acidic phospholipase A2 from Indian saw-scaled viper (Echis carinatus) at 2.6 A resolution reveals a novel intermolecular interaction. *Acta Crystallogr. D Biol. Crystallogr.* **2004**, *60* (Pt 1), 66-72. https://doi.org/10.1107/s090744490302208x.

(13) Waterhouse, A. M.; Procter, J. B.; Martin, D. M.; Clamp, M.; Barton, G. J. Jalview Version 2—a multiple sequence alignment editor and analysis workbench. *Bioinformatics* **2009**, *25* (9), 1189-1191. https://doi.org/10.1093/bioinformatics/btp033.
